# Estimates of molecular convergence reveal multiple genes with adaptive variation across teleost fish

**DOI:** 10.1093/molbev/msag015

**Published:** 2026-01-28

**Authors:** Agneesh Barua, Malvika Srivastava, Brice Beinsteiner, Vincent Laudet, Marc Robinson-Rechavi

**Affiliations:** Department of Ecology and Evolution, University of Lausanne, Lausanne, Switzerland; Evolutionary Bioinformatics, Swiss Institute of Bioinformatics, Lausanne, Switzerland; Department of Ecology and Evolution, University of Lausanne, Lausanne, Switzerland; Helmholtz Pioneer Campus, Helmholtz Zentrum München, Neuherberg, Germany; Marine Eco-Evo-Devo Unit, Okinawa Institute of Science and Technology Graduate University, Onna son, Okinawa, Japan; Marine Research Station, Institute of Cellular and Organismic Biology (ICOB), Academia Sinica, 23-10, Dah-Uen Rd., Jiau Shi, I-Lan 262, Taiwan; Department of Ecology and Evolution, University of Lausanne, Lausanne, Switzerland; Evolutionary Bioinformatics, Swiss Institute of Bioinformatics, Lausanne, Switzerland

**Keywords:** molecular convergence, teleost fishes, protein evolution

## Abstract

Molecular convergence, where specific nonsynonymous changes in protein-coding genes lead to identical amino acid substitutions across multiple lineages, provides strong evidence of adaptive evolution. Detecting this signal across diverse taxa can reveal adaptive variation that may not be apparent when studying individual lineages. In this study, we search for convergent substitutions in the most speciose group of vertebrates, teleost fishes. Using an unsupervised approach, we detected convergence in 89 protein-coding gene families across 143 chromosomal-level genomes. To assess their functional implications, we integrate data on protein properties, gene expression across species and tissues, single-cell RNA sequencing of zebrafish embryonic development, and gene perturbation experiments in zebrafish. We found that, on average, the convergent genes had more gene copies as compared to background sets of genes. The convergent genes were associated with diverse processes including embryonic development, tissue morphogenesis, metabolism, and heat stress response. We found evidence that convergent substitutions were more radical than nonconvergent substitutions. When analyzing the expression of the convergent genes, we found that only one-third of them were tissue-specific, while the majority were expressed across multiple tissues and cell types. Genetic perturbation data further showed that the convergent genes can affect multiple structures across diverse tissues. These results highlight the important functional roles of the convergent genes, their potential pleiotropic nature, and suggest that they may underlie the evolution of lineage-specific adaptations in teleost fishes.

## Introduction

Evolutionary convergence, characterized by different lineages independently acquiring similar character states, in response to similar ecological challenges, is pervasive in nature (Rosenblum et al. 2014). While convergence typically invokes images of recurring phenotypic traits, convergence can also occur at the molecular level, where the same amino acid substitution can occur at the same site in orthologous proteins (Storz 2016; Rey et al. 2019). Identifying molecular signatures of convergence is integral to understanding the genetic basis behind the evolution of phenotypes, as well as aiding in recognizing adaptive developmental processes across diverse groups of animals (Steppan et al. 2002; Losos 2011). Indeed, studies of molecular convergence have provided insights into the origin of traits like echolocation in birds and mammals, toxin resistance in beetles, and carnivory in plants, just to name a few (Dobler et al. 2012; Fukushima et al. 2017; Sadanandan et al. 2023). The earliest methods to estimate molecular convergence relied on reconstructed ancestral sequences to identify sites in foreground branches that had the same amino acid substitution but differed in their ancestral sequence (Zhang and Kumar 1997; Zou and Zhang 2015). Later methods refined this approach by estimating ratios of repeated and divergent changes in amino acids in foreground branches to distinguish true instances of repeated substitutions from null expectations (Castoe et al. 2009; Thomas and Hahn 2015). However, requiring foreground lineages to share the same amino acid substitution can limit the detection of functionally convergent changes that arise through different amino acid substitutions (Zhen et al. 2012; Mohammadi 2022). Additionally, restricting analyses to a predefined set of foreground branches based on an a priori phenotype of interest may overlook other potential adaptive changes. To capture a broader range of repeated evolution, studies would benefit from unbiased analytical approaches.

Studies have consistently identified certain gene categories—such as gas transporters, sensory proteins, and xenobiotic-processing enzymes—as frequent targets of adaptation to new environments (Martin and Orgogozo 2013). The prevalence of evolutionary convergence, especially in certain gene categories, highlights a potential deterministic aspect of the evolutionary process (Blount et al. 2018). In this study, we use a data-driven unsupervised approach to identify instances of repeated molecular convergence in protein-coding orthologs across 143 teleost fish species. Teleost fishes represent the most specious group of vertebrates on Earth, encompassing a wide array of phenotypes, morphologies, habitats, and life histories (Streelman et al. 2007; McMenamin and Parichy 2013). This diversity positions teleosts as an ideal group for exploring the broad evolutionary processes giving rise to species diversity and richness. Furthermore, the availability of high-quality genomes for numerous species, coupled with single-cell sequencing and functional data from zebrafish (*Danio rerio*), offers an excellent opportunity to investigate the potential effects variations in coding sequence might have on tissue functionality and animal phenotypes (Lange et al. 2024).

## Results

### Data Collection and Orthology Analysis

**Figure 1:**
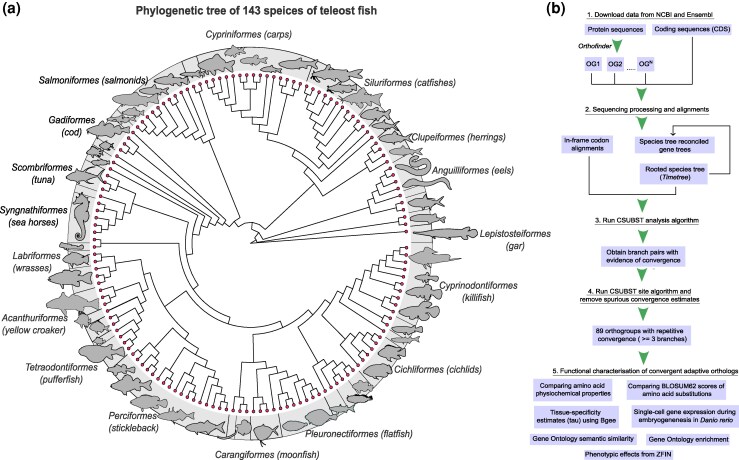
Phylogenetic tree of species and workflow schematic. a) Phylogenetic tree of 143 teleost fish species used in the study. The silhouettes are not to scale and are representative of species in each order. For improved clarity, only a few orders are labeled. For a full list, refer to Table S1. The time-calibrated, rooted species tree was obtained from timetree.org (Kumar et al. 2022) (Figure S13). b) Schematic describing the workflow of this study. The silhouettes are obtained from phylopic.org.

We selected teleost fish species that had chromosomal-level annotated genomes from the National Center for Biotechnology Information (NCBI) (Sayers et al. 2022) and Ensembl genome databases (Martin et al. 2023). Our final list of genomes comprised 143 species from 40 orders (most sampled were *Perciformes* with 17 species) covering 85 families (most sampled were *Salmonidae* with 10 species) (Fig. 1a and Figure S13, Table S1). We use the spotted gar, *Lepisosteus oculatus*, a nonteleost that did not undergo the teleost-specific whole genome duplication (Braasch et al. 2016) as our outgroup. Using OrthoFinder (Emms and Kelly 2019), we estimated orthologous relationships across species (Fig. 1b). Orthologs represent protein-coding genes in different species that diverged from a common ancestor following a speciation event, and sets of orthologous genes are placed in orthogroups as inferred by OrthoFinder. The analysis resulted in 40,940 orthogroups containing over 4 million protein-coding orthologs. A total of 1,723 orthogroups contained all the species. To ensure that we focus on genes that reflect the evolution of teleost fish diversity, we selected orthogroups with at least one species from each of the 40 orders in our dataset. Additionally, to keep computational times reasonable, we restricted our analysis to orthogroups containing a maximum of 1,500 genes. This resulted in a final dataset of 9,273 orthogroups with around 2 million orthologous genes (Dataset S1 in online repository), i.e. more than half of all the genes in the original 40,940 orthogroups. Due to this filtering, we focus on a more conserved set of genes across taxa. While lineage-specific genes can experience different evolutionary trajectories, leading to the evolution of specific (or even the same) adaptations in multiple lineages, the focus of our study is repeated evolution using the same genetic building blocks. We annotated and functionally characterized the excluded orthogroups and found that they were mostly associated with immune processes and ion transport, whereas the gene sets we used in our analysis were involved in many diverse processes (Figures S1 and S2). Although processes related to ion transport and immunity are important, our sample encompassed a diverse array of processes and provided a powerful window into teleost evolution.

### How many genes have evolved convergent substitutions in teleosts?

We obtained estimates for molecular convergence using the CSUBST (Combinatorial SUBSTitutions) algorithm introduced by Fukushima and Pollock (Fukushima and Pollock 2023). This algorithm allows for exploration of genomic datasets to identify repeated amino acid substitutions without specifying any foreground lineage or any predefined hypothesis regarding which traits can be considered as adaptations. It combines a pairwise exhaustive search across all possible branch combinations on a phylogeny, followed by a heuristic step that examines branch combinations in more than 2 independent lineages (Fukushima and Pollock 2023). CSUBST draws inspiration from the widely used d*N*/d*S* ratio (parameterized as *ω*), which measures the rate of protein evolution as a ratio between nonsynonymous and synonymous substitution rates (Yang 2014). Fukushima and Pollock (2023) developed a similar metric, *ω*_C_, which applies to substitutions repeatedly occurring on combinations of separate phylogenetic branches. This metric is designed to assess the relative rates of convergent evolution by comparing the rates of nonsynonymous convergence and synonymous convergence across a range of distinct phylogenetic branches. The rationale behind this approach is that truly adaptive functional changes to proteins are most frequently caused by nonsynonymous codon substitutions that alter the amino acid sequence. If distantly related lineages exhibit the same amino acid change at the same position this is a strong signal for adaptive evolution as the emergence of such repeated substitution by neutral evolution or simple molecular constraints alone is extremely low. It is important to note that the convergence signal in CSUBST is determined by comparison against a neutral expectation. Similar to how a *ω* (d*N*/d*S*) > 1 indicates positive selection, the convergence ratio *ω*_C_ (d*N*_C_/d*S*_C_) > 1 represents directional convergence beyond neutrality. CSUBST generates estimates for various types of combinatorial substitutions. These include divergent changes, characterized by substitutions from any ancestral amino acid to a different extant amino acid, and convergent changes, which involve substitutions from any ancestral amino acid to a specific amino acid occurring at the same position across multiple lineages.

**Figure 2:**
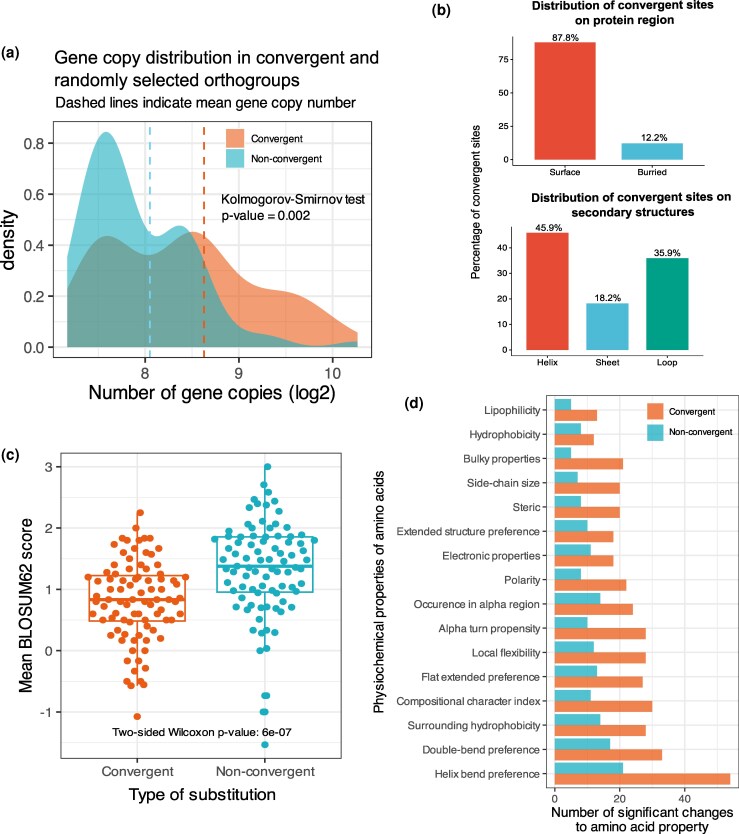
Gene copy number of convergent orthogroups and changes to physicochemical properties following amino acid substitutions. a) The convergent orthogroups had a higher distribution of genes with high copy number as compared to the background of randomly selected genes. b) A majority of the convergent sites were on the external surface of the protein, with most of them located on the helices of proteins. c) Difference in mean BLOSUM62 score during a change from an ancestral amino acid to extant amino acid for the 2 types of protein substitutions: convergent and nonconvergent. The plot shows that the convergent substitutions on an average had lower BLOSUM62 scores as compared to other, nonconvergent substitutions occurring within each protein. d) Comparison of changes in physicochemical properties of ancestral and extant amino acids for convergent and nonconvergent substitutions. The panel shows that the convergent substitutions had a higher number of statistically significant changes to amino acid properties as compared to the nonconvergent substitutions.

We estimated *ω*_C_ for all independent branch pairs across 9,273 orthogroups and classified branch pairings that jointly satisfied minimum thresholds of 5 for *ω*_C_ and for observed nonsynonymous convergence (OCN) (*ω*_C_ ≥ 5 & OCN ≥ 5) as evidence for convergence. These thresholds were based on validations in the original paper (Fukushima and Pollock 2023). We only focused on convergence that was observed in 3 or more branch combinations (Table 1). Next, using the CSUBST *site* function, we mapped convergent amino acid substitutions on predicted protein structures. Following a final post-processing step to remove certain instances of spurious convergence (Figure S3) (see Materials and Methods), the final output comprised 89 orthogroups (274 genes), each showing evidence for repetitive convergence in multiple independent branches across teleosts (Table 1). We checked for any potential bias of the OCN metric to estimate convergence for longer genes or orthogroups containing more genes simply because the sequence space is larger. Using a Pearson correlation test, we found no significant relationship between gene number or gene length and the OCN metric (Figure S4). We also compared the distribution of gene copy numbers within the convergent orthogroups and a background of randomly selected nonconvergent orthogroups. Interestingly, we found that on average the convergent orthogroups had higher copy number than random sets of orthogroups (5,000 bootstrap of 1-sided Kolmogorov–Smirnov test; average Benjamini–Hochberg corrected, *P* = 0.002) (Fig. 2a). Beyond this global pattern, we observed similar trends within individual clades. At the order level, 86% (31 out of 36) of clades showed evidence of higher copy numbers among convergent genes, and at the family level, 83% (61 out of 73) showed the same pattern. Thus, while the majority of clades had higher copy numbers in convergent orthogroups, this trend was not universal across all teleost lineages (Figure S5).

**Table 1:** Convergence statistics obtained from CSUBST.

Arity	*N*	OmegaC	OCN	*dNc*	*dSc*
3	85	129.6	9.9	52.1	0.33
4	2	Inf	10	3,677.3	0
5	1	Inf	5.3	20,230.8	0
7	1	1.4e+10	5.7	427e+5	0.29

Arity is the number of combinatorial branches for which convergence was found. *N* is the number of orthogroups per arity with evidence of convergence. OmegaC median $\left(\omega_{c}\right)$ value. *dNc* is the median value for the number of nonsynonymous convergent substitutions. *dSc* is the median value for the number of synonymous convergent substitutions.

Most convergence involved 3 branches, with a few cases involving 4, 5, or 7 branches (Table 1). The orders *Perciformes*, *Acanthuriformes*, and *Cyprinodontiformes* had the most convergent orthogroups, as did the families *Cichlidae*, *Percidae*, and *Tetraodontidae* (Figure S6). The taxonomic distribution of convergent orthogroups was different from that simply expected by sampling (5,000 bootstrapped 2-sided Kolmogorov–Smirnov test, *P* < 0.05) (Figure S6). In terms of ecological characteristics (based on available data from FishBase), the highest proportion of convergent orthogroups was estimated in tropical sea fish (Figure S7). However, we could not infer any overall significant relationship between convergence in a specific orthogroup and ecological features such as climate, ecosystem type, or food items. While this may partly reflect the low granularity of the categories, the convergent substitutions could also represent highly specific adaptations that are not captured by such broad classifications, highlighting the need for more detailed and targeted categorization.

### Estimating functional consequence of amino acid substitutions

Mapping of convergent substitutions onto the protein structure revealed that most convergent sites were located on the surface residues of the protein, potentially enabling interaction with other proteins (Fig. 2b). However, convergent sites were not significantly overrepresented on the surface residues (chi-square test, *P* = 0.941). This implies that although most convergent sites were on the protein surface, so were most other sites. In terms of secondary structure, most of the convergent sites were located on helices, but were not overrepresented (chi-square test, *P* = 0.567). The heterogeneity of the protein structure's resolution likely reduces the statistical power of these comparisons (see Materials and Methods).

To get an estimate of the functional consequences of the convergent substitutions, we compared the average BLOSUM62 scores of the convergent and nonconvergent substitutions occurring within each of the proteins with convergent substitutions. The BLOSUM62 score measures how frequently one amino acid is replaced by another in evolution. Higher scores indicate common, conservative substitutions with little functional impact, while lower scores reflect rare changes that are more likely to alter protein structure or function. We found a significant difference (2-sided Wilcoxon rank sum test, *P* = 6 × 10^−7^) in BLOSUM62 scores between convergent and nonconvergent substitutions (Fig. 2c). On average, the convergent substitutions had lower scores, implying that these changes are rare in evolution and have a higher probability to alter protein function.

**Figure 3:**
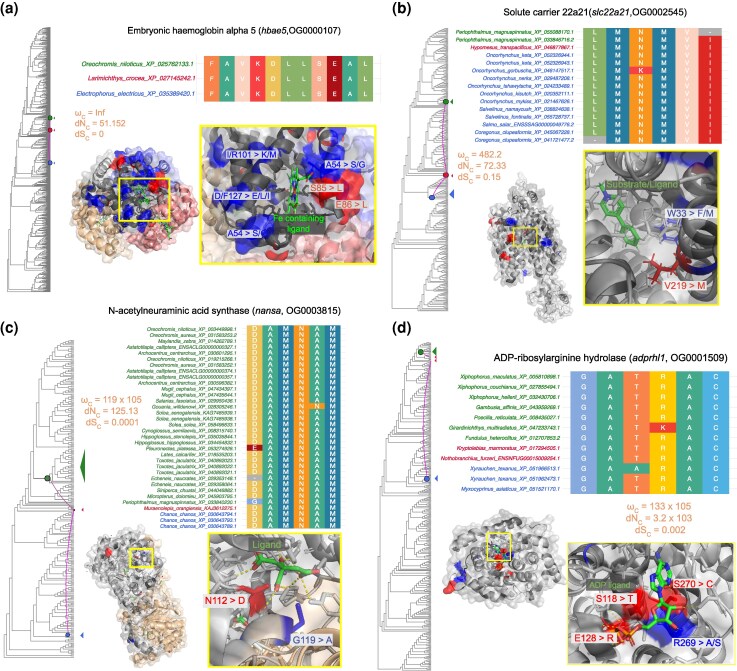
Potential cases of site-specific functions. The red sites represent convergent sites, while the blue represent divergent sites. The amino acid letter and site followed by the arrow represent the change in sequence at that particular position on the protein structure. For example, in *hbae5*, S85 > L means a serine at position 85 on the protein structure changed to a leucine. These sites are localized in important ligand (green) binding sites. The phylogeny is a species-reconciled gene tree. The colored dots represent the branches in which convergence was found. The species names and colors correspond to the extant species descended from these branches. The alignment shows each of the aligned sites in the species with convergent substitutions. These sites are different from those in other species (full alignment in Dataset S8). Convergence metrics for each branch combination are also shown.

Next, we compared the changes in physicochemical properties of amino acid residues resulting from convergent substitution and nonconvergent substitutions within each protein. We compared 17 different amino acid properties and found that 95% of the convergent orthogroups (85 out of 89) had significant changes (2-sided Wilcoxon rank sum test, Benjamini–Hochberg corrected *P* < 0.05) in at least one of the 17 physicochemical properties analyzed (Fig. 2d). These types of amino acid replacements leading to changes in physicochemical properties tend to have a stronger effect on protein structure or function and are classified as “radical” amino-acid changes (Hanada et al. 2007). The number of significant changes was higher for the convergent substitutions than for the nonconvergent ones, confirming that they are radical. The most frequent convergent changes affected helix bend preferences, which align with the observation of their higher distribution on protein helices. However, the highest proportions of significant convergent change were in bulky properties, side-chain size, and alpha turn propensity (0.8, 0.74, 0.73, respectively). The lowest proportions were in occurrence in alpha region, electronic properties, and hydrophobicity (0.63, 0.62, 0.6, respectively). A full description of the properties and their potential effects can be found in Table S2. In certain proteins, the convergent substitutions were localized on functionally important sites (Fig. 3). We discuss the potential functional implication of these substitutions in the discussion. Overall, we observed that nonsynonymous convergent substitutions at the codon level were rare and led to amino acid changes with different chemical properties.

We also used the Fast Unconstrained Bayesian Approximation (FUBAR) (Murrell et al. 2013) to estimate per-site synonymous and nonsynonymous rates and found that over 80% of the convergent sites showed evidence of purifying selection across all the convergent genes, while only 1% showed evidence for diversifying selection. Around 19% of the sites did not provide a reliable estimate for either selection regime (Figure S8).

### Which biological processes are the convergent genes involved in?

**Figure 4:**
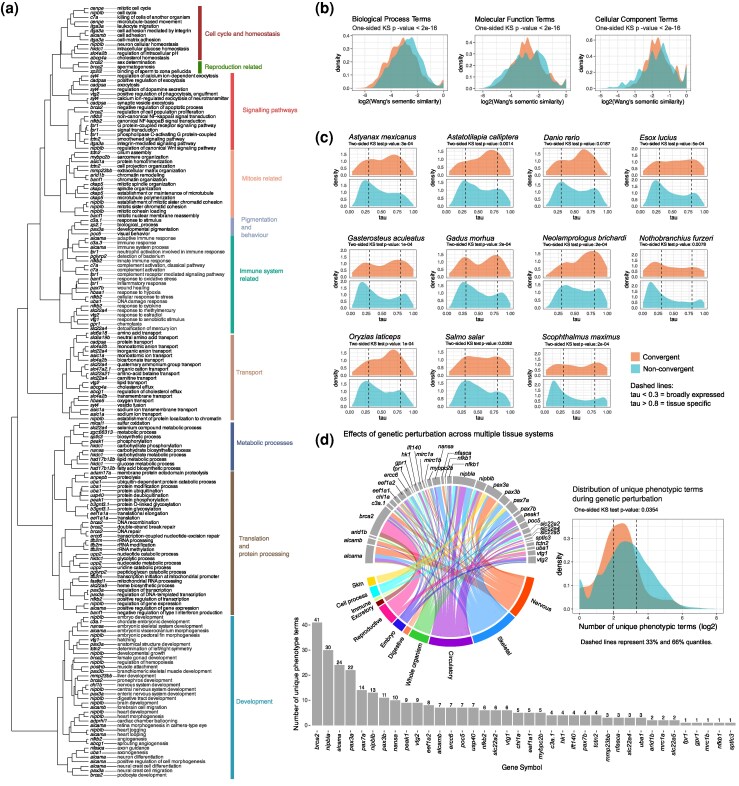
Multiple lines of evidence showing the functional effects of the convergent genes. a) Dendrogram of convergent *D. rerio* orthologs based on GO semantic similarity. The dendrogram shows the different genes and their associated Biological Process GO descriptions, categorized into 10 different clusters. Full plots along with plots for other GO categories can be found in Dataset S12. b) Distribution plots showing that the GO terms of the convergent genes tend to have lower semantic similarity scores as compared to a background set of random genes. c) To estimate tissue specificity, we use the tau index. This index indicates how specific or broadly expressed a gene or transcript is within studied tissues. The convergent genes had intermediate levels of tissue specificity, i.e. most of the genes had tau values between broadly expressed (tau < 0.3) and tissue-specific (tau > 0.8). d) Using data from ZFIN, this panel examines the unique phenotypic terms associated with genetic perturbation of convergent orthologs in *D. rerio*. The histograms show the number of unique phenotypic terms associated with genetic perturbation in *D. rerio.* These data are obtained from various studies and show that the convergent orthogroups can have multiple phenotypic effects. The chord diagram shows that for some genes, the perturbation phenotypes occur across multiple tissue systems (e.g. *alcama*, *pax3a*). The length of the gray bars corresponds to the frequency values on the histogram. Data used to construct the chord diagram can be found in Table S6. The density plot shows that compared to the background level or all genes with phenotypic information in ZFIN, the convergent genes affect an intermediate number of phenotypes.

We performed a Gene Ontology (GO) enrichment to get a general overview of the biological processes associated with these convergent orthogroups. The analysis revealed a significant enrichment (Hypergeometric test, Benjamini–Hochberg corrected *P* < 0.05) for terms predominantly linked to biomolecule metabolism, response to hormones or stimuli such as heat, and processes of embryonic development and tissue morphogenesis (Figure S2). Interestingly, we also identified enriched GO terms related to xenobiotic-processing (GO:0071466-response to xenobiotic stimulus), gas transport (GO:0015671-oxygen transport), and sensory system development (GO:0060042-retinal morphogenesis in camera-type eye), which were reported to be frequent targets of adaptation to new environments (Fig. 4a) (Martin and Orgogozo 2013). We also analyzed the semantic similarities of GO terms between convergent orthogroups and a background set to quantify the extent of functional diversity, providing an estimate of the multifunctional nature of the convergent genes. The higher the semantic similarity of GO terms, the more closely related are aspects of a gene's function, while a lower semantic similarity implies higher functional diversity. A critical requirement for accurate semantic similarity analyses is well-annotated GO terms, based on both experimental and in silico evidence (Pesquita 2017). Therefore, we carried out this analysis with the *D. rerio* orthologs. We found that GO terms of the convergent orthogroups, on average, exhibited lower semantic similarity than the background (5,000 bootstrapped 1-sided Kolmogorov–Smirnov test, *P* < 10^−16^) (Fig. 4b). This suggests that they are associated with a diverse range of biological processes (Pesquita 2017; Yu 2020). This pattern was consistent across GO term categories (Fig. 4b).

To better resolve the function of these convergent orthogroups, particularly in the context of development and tissue formation, we carried out a comprehensive analysis of gene expression across species and developmental transitions. Using gene expression data from the Bgee database (Bastian et al. 2025), we calculated tissue specificity of the convergent orthogroups in 11 fish species (see Materials and Methods) and across 8 different tissue types (brain, eye, heart, liver, muscle, ovary, skin, and testis). Approximately one-third (28 out of 89) of the convergent orthogroups were tissue-specific, with the highest number in the liver (Figure S9). Overall, we observed that the proportion of convergent orthogroups that are tissue-specific is no different from the proportion of tissue-specific genes in our dataset as a whole (2-sided Wilcoxon signed rank test, *P* = 1), suggesting that the convergent orthogroups are not more likely to be tissue-specific, and that we aren’t oversampling tissue-specific genes. However, on a tissue-by-tissue basis, we found a significant difference in the convergent/nonconvergent ratio between tissue-specific and nontissue-specific genes only for the liver and not for other tissues (Fisher's exact test, *P* < 0.05); in other words, our convergent orthogroups set had a higher proportion of liver-specific genes than expected by chance. Of note, our estimates of tissue specificity were limited to adult fish.

Next, we compared the distribution of tissue specificity of the convergent orthogroups to all other genes in each of the 11 species of fish from Bgee. In contrast to the previous analysis, where we checked whether convergent orthogroups were enriched for tissue-specific genes compared to other genes in the genome, this analysis compares the distributions of the values of the tissue-specificity index. The distribution of tissue specificity of the convergent orthogroups was significantly different compared to the background (5,000 bootstrapped 2-sided Kolmogorov–Smirnov test, *P* < 0.05), with the convergent orthogroups having a higher frequency at intermediate values of tissue specificity (Fig. 4c).

Lastly, we collected data from the Zebrafish International Network (ZFIN) database (Bradford et al. 2022) to examine the distribution of unique phenotypic terms associated with genetic perturbation (knockdowns, mutation screens, and morpholino experiments) in *D. rerio*. The ZFIN database provides a wide array of expertly curated cross-referenced genetic and genomics data that is routinely used to study gene functions (Bradford et al. 2022). For the convergent orthogroups with data in ZFIN, we observed that genetic perturbation can affect several structures across multiple tissue systems (Table S4) (Fig. 4d). Compared to the background of other genes with available phenotypic data, the convergent orthogroups had a higher distribution at intermediate levels of affected phenotypic terms, significantly different from the background (5,000 bootstrapped 1-sided Kolmogorov–Smirnov test, *P* = 0.0354) (Fig. 4d).

### Single cell expression dynamics of convergent genes during embryonic development

**Figure 5:**
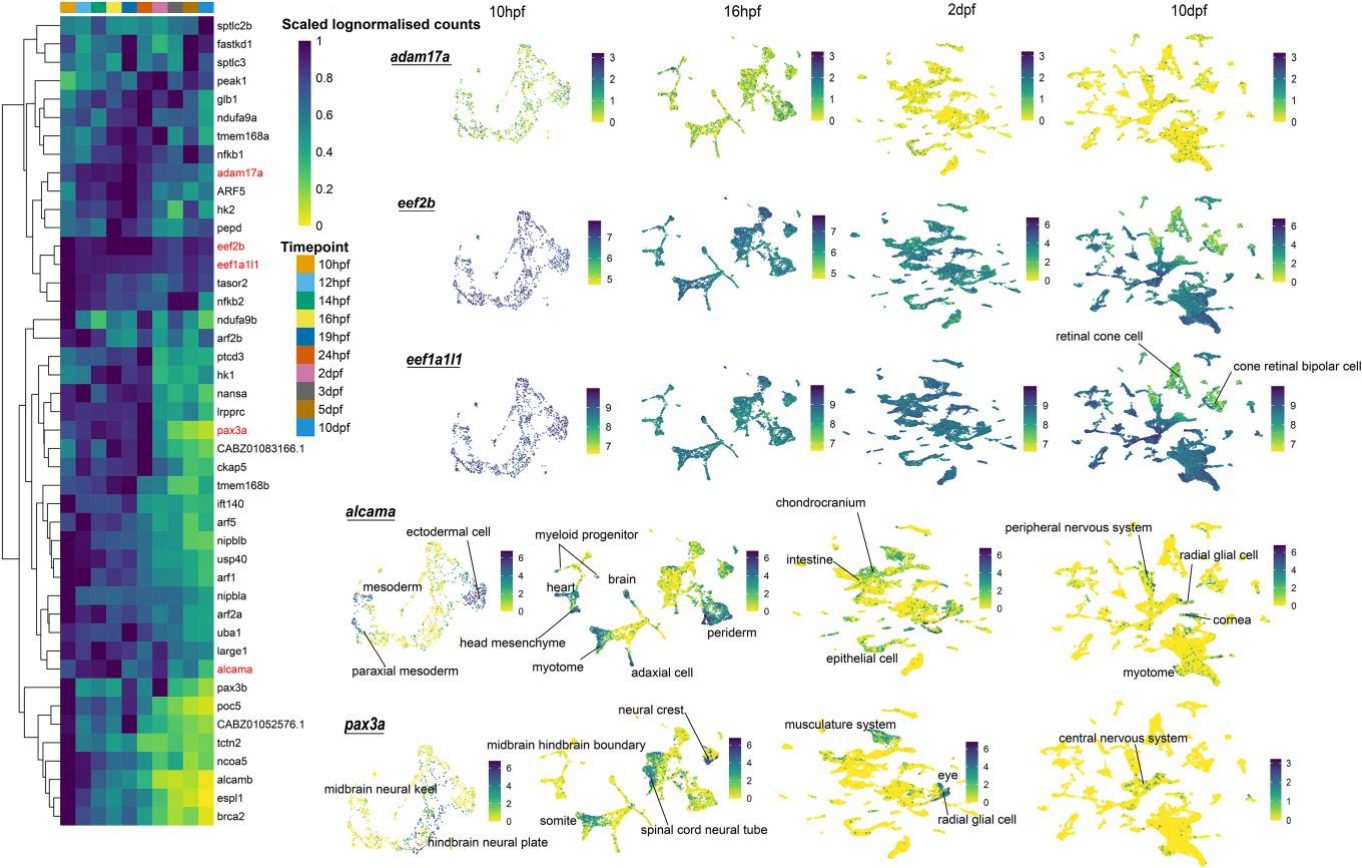
Convergent genes expressed in multiple cell types throughout embryonic development in zebrafish. Single-cell RNA sequencing shows that the convergent genes expressed throughout embryonic development can be divided into 3 groups. This plot shows 2 groups: genes with ubiquitous expression throughout embryonic development and across multiple cell types, and genes expressed throughout embryonic development but in specific cell types. The heatmap shows expression of genes throughout zebrafish embryonic development, while the UMAPs show expression across cell types at specific time intervals. The darker the color, the higher the expression. The genes in red on the heatmap correspond to genes in the UMAPs. Genes like *adam17a*, *eef2b*, and *eef1a|1* have expression in almost all cell types. While genes like *alcama* and *pax3a* show more restricted expression in specific cell types. A key feature of both these groups is that the genes are expressed in multiple tissue systems (e.g. heart, musculature, nervous system). The color bar in the UMAP is log-normalized counts.

To gain insight into the expression dynamics of the convergent orthogroups during development, we analyzed single-cell RNA sequencing data for embryonic development in zebrafish (*D. rerio*) from the Zebrahub atlas (Lange et al. 2024). We identified 3 groups of genes from the convergent orthogroups based on their expression patterns. First, genes with ubiquitous expression throughout embryonic development and across multiple cell types (Fig. 5). These included genes such as *adam17a* (ADAM metallopeptidase 17a; OG0003642), *eef2b* (eukaryotic translation elongation factor 2b; OG0001781), and *eef1a1|1* (eukaryotic translation elongation factor 1 alpha 1; OG0000135) (complete list in Table S3). Involved in the Notch signaling pathway, *adam17a* showed stable expression in the early stages at 10 hpf (hours post-fertilization), 16 hpf, and 2 dpf (days post-fertilization) but declined at 10 dpf. Both *eef2b* and *eef1a1|1* showed high expression levels across development, with a slight reduction in eye-related cell types at 10 dpf.

The second group comprised genes expressed throughout embryonic development but in specific cell types. Examples include *alcam-a* (activated leukocyte cell adhesion molecule a; OG0001823) and *pax3a* (paired box 3a; OG0000460) (Fig. 5). *Alcam-a* was highly expressed in cell types related to lens placode, neural crest, radial glial cell, and cornea (Fig. 5; 10 dpf) (Diekmann and Stuermer 2009; Murayama et al. 2023). With high expression in cell types related to somites, spinal cord neural tubes, radial glial cells, neural crest cells, and the central nervous system, the expression of *pax3a* aligned with its known role in eye development and pigmentation (Nelms 2010).

**Figure 6:**
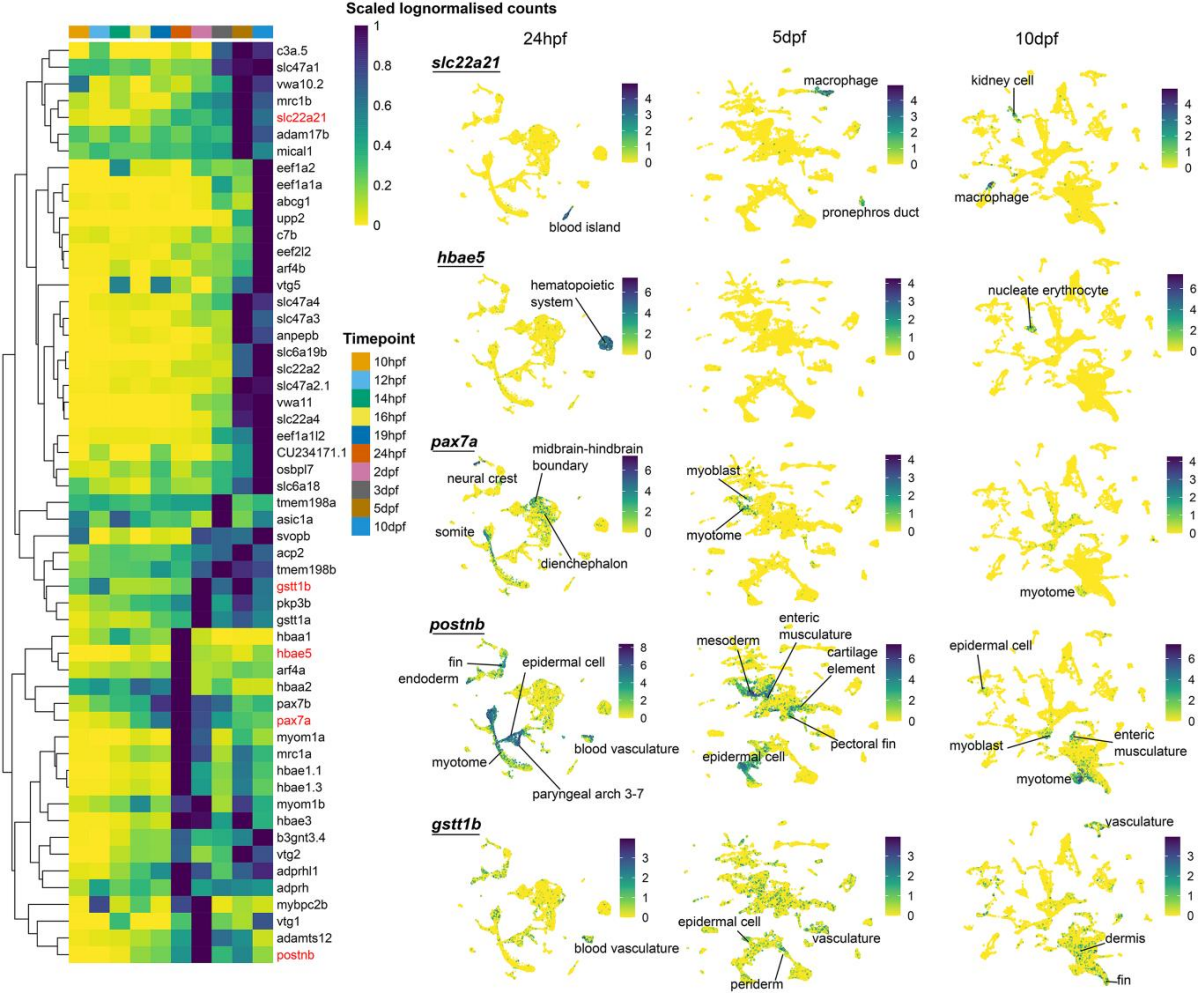
Convergent genes expressed in multiple cell types at specific stages of embryonic development in zebrafish. In contrast to the previous plot, these sets of convergent genes display highly restricted ontogenic expression patterns, being expressed only at specific timepoints. These genes are also expressed in multiple cell types. The genes in red on the heatmap correspond to genes in the UPAMs. The color bar in the UMAP is log-normalized counts.

The third group of genes was predominantly expressed late in embryonic development (Fig. 6). Genes in this group, such as *slc22a21* (OG0002545) and *hbae5* (OG0000107), were predominantly expressed between 24hpf and 10dpf (Fig. 6). *Slc22a21* was associated with the excretory and vascular systems, while *hbae5* was restricted to hematopoietic cells. Other genes, such as *postnb* (periostin, osteoblast-specific factor b; OG0002859) and *gstt1b* (glutathione S-transferase theta b, OG0004038), have roles in the formation of the extracellular matrix of the skin (Snider et al. 2009) and liver detoxification, respectively.

## Discussion

### Potential cases of site-specific functions

In cases where it was possible to identify site-specific annotations from X-ray diffraction or cryo-electron microscopy structures, we found convergent substitutions at critical functional sites. Although specific experiments are needed to validate their function, we can make several predictions based on empirical evidence from previous studies.

For instance, in embryonic hemoglobin alpha 5 protein (*hbae5*; OG0000107), we observed both convergent and divergent substitutions located around the protoporphyrin IX containing Fe ligand binding site (Fig. 3a). Changes in the hemoglobin protein, particularly alterations at the binding site of the Fe ligand have been linked to increased tolerance to acute hypoxia in vertebrates (Weber 2007; Ragsdale et al. 2022). This suggests a role of these convergent substitutions in increased tolerance to hypoxic conditions. The convergent substitutions were observed in *Electrophorus electricus* (electric eel), *Larimichthys crocea* (yellow croaker), and *Oreochromis niloticus* (Nile tilapia). Electric eel and Nile tilapia can both resort to air-breathing during times of hypoxia (Farber and Rahn 1970; Obirikorang et al. 2020), while the yellow croaker, which is often found in muddy estuaries and coastal waters, has evolved protective, hemoglobin-containing mucous that aids in oxygen transport during hypoxia and air exposure (Ao et al. 2015). The convergent substitutions in *Hbae5* could be linked to these adaptations.

We also detected convergent substitutions within the interior of the channel of the barrel-like structure of the solute-carrier protein *slc22a21* (OG0002545), near the 1-methyl-4-phenylpyridinium ligand binding site (Fig. 3b). Given the role of *slc22a21* in maintaining the homeostasis of various organic ions, these substitutions could impact processes related to the absorption, distribution, metabolism, and excretion of biomolecules (Mihaljevic et al. 2016). Members of the *Salmonidae* family, as well as *Periophthalmus magnuspinnatus* (mudskippers), and *Hypomesus transpacificus* (delta smelt), which harbor the substitutions, all experience varying levels of salinity throughout their life cycles (Parry 1960; You et al. 2018; Hung et al. 2022). The convergent substitutions could have helped these species adapt to changes in salinity. We also observed expression of *slc22a21* in the liver and pyloric caecum across multiple larval stages of *Salmo salar*, with a subsequent expression in the kidney in the post-juvenile stages (Figure S10). Moderate expression was also observed in muscle tissue, the brain, and eye, corroborating its known functional activity in tissue systems such as the liver, kidneys, skeletal system, and central nervous system (Nigam 2018).

The orthogroup OG0003815 encodes N-acetylneuraminic acid synthase (*nansa*). Multiple lines of evidence suggest that *nansa* plays a crucial role in skeletal development, particularly in head formation. Knockdown of *nansa* in zebrafish leads to abnormal skeletal development, while microinjection of *nansa* morpholino into zebrafish embryos results in a small head size and developmental anomalies. Additionally, mutations in the human ortholog NANS, which alter its enzymatic activity, have been linked to skeletal dysplasia (van Karnebeek et al. 2016). These results support the gene's essential role in skeletal formation, particularly in the development of the head. The convergent substitutions are present at multiple sites on the protein surface, including a substitution within the protein's interior at a functional ligand-binding site (Fig. 3c). The species within this orthogroup display different body shapes, ranging from the fusiform body shapes of *O. niloticus*, *Astatotilapia calliptera* (eastern river bream), and *Toxotes jaculatrix* (banded archerfish), to the elongated (somewhat anguiliform) body shapes of *Salaris fasciatus* (jeweled blenny), *Muraenoplis orangiensis* (Patagonian morray cod), and *Gouania willdenowi* (blunt-snouted clingfish), as well as the depressiform body shapes of *Soela solea* (common sole) and *Hippoglossus stenolepis* (Pacific halibut). Interestingly, the remora, which has evolved a flat oval sucking disk (modified dorsal fin) used to attach to larger marine animals like sharks, also harbors substitutions in the *nansa* gene. This gene was also highly expressed in the adult anal fin, skin, and eye of *A. calliptera*, as well as in the spleen during the early juvenile stages (Figure S10). The high expression in the skin and anal fin is consistent with its known role in skeletal development (van Karnebeek et al. 2016). The occurrence of convergent substitutions appearing in organisms with divergent phenotypes has been observed in marine mammals as well; for instance, substitutions in the *S100A9* and *MGP* genes were linked to both high and low bone density occurring in shallow diving manatees and deep diving dolphins, respectively (Foote et al. 2015). Therefore, the convergent substitutions in the *nansa* gene likely influenced the sialylation of glycoproteins, which altered downstream pathways affecting head and skeletal morphology.

Finally, ADP-ribosylarginine hydrolase (*adprhl1*; OG0001509) exhibited convergent substitutions at the adenosine-5′-diphosphate ligand site (Fig. 3d). This active site is important for the post-translational modifications of proteins (Mashimo et al. 2014). Although the ADP-ribosylarginine hydrolase gene family affects multiple processes, its link to tumorigenesis might be particularly relevant for the *Xiphophorus* (platyfish) species, which readily form skin melanomas (Kozlov 2014; Mashimo et al. 2014).

In addition to the above cases of site-specific molecular convergence, we also observed a remarkable convergence in the polypeptide N-acetylgalactosaminyltransferase 6 (*GALNTL6*) along 7 branches, primarily in Pomacentridae and Syngnathidae (Figure S11). *GALNTL6* is proposed to play a role in the initiation or regulation of mucin-type O-glycosylation (Diamant et al. 2025). Using data from Bgee, we observed high expression in the reproductive and skeletal systems across teleost species (Figure S11). Although its precise enzymatic activity remains poorly characterized, the expression patterns suggest that *GALNTL6* may have lineage-specific functional roles in reproduction and skeletal development, warranting further investigation.

### Pleiotropy and adaptive evolution

Considering that many of the convergent genes have multiple functions and are expressed in multiple tissues (as demonstrated by their low GO terms semantic similarity and low tissue specificity), they could have beneficial pleiotropic effects during periods of ecological shifts (Bomblies and Peichel 2022; Rennison and Peichel 2022; Kinsler et al. 2024; Barua et al. 2025). Although traditionally considered as a source of genetic constraints (Orr 2000), studies in several model systems as well as natural populations have shown that the idea of pleiotropy constraining adaptation needs to be reconsidered (Paaby and Rockman 2013; Zhang 2023). For example, a vital gene responsible for the adaptation to freshwater habitats in sticklebacks, Ectodysplasin (*Eda*), is highly pleiotropic and responsible for ectodermal appendage formation, sensory system patterning, and schooling behavior (Mills et al. 2014; Greenwood et al. 2016). Intermediate levels of pleiotropy might even enhance adaptive potential by causing greater fitness gain and increasing the fixation of beneficial alleles (Hansen 2003; Wang et al. 2010; Barua et al. 2025).

In a recent experimental evolution study in yeast, Kinsler et al. (2024)) showed that large-effect adaptive mutations are generally pleiotropic, improving both respiration and fermentation, and tend to occur during the early stages of evolution. Although they observed a general shift from pleiotropic to modular adaptation, they did observe some strongly adaptive yeast clones that continued to improve in both respiration and fermentation, causing them to achieve high levels of fitness. The authors also discuss observations in multiple studies, which show that when yeast evolved in a glucose-limited environment, the first adaptive mutations were always in pleiotropic genes that enhance both respiration and fermentation. Therefore, evolution along the axes of pleiotropic genes might be a feature of evolution during the initial periods following ecological shifts. We can also use this hypothesis to explain the patterns we observe in our study.

An ecological shift might have resulted in the ancestors of salmonids, mudskippers, and delta smelt to each experience varying levels of salinity. The varying levels of salinity could also impact the available food resources as food webs differ between marine and freshwater habitats. To adapt to the new environmental conditions, changes in the *slc22a21* gene might prove particularly beneficial. Along with maintaining the homeostasis of organic ions between tissues and the interfacing body fluids, this gene family can impact tissue systems such as the liver, kidneys, skeletal system, and central nervous system (Nigam 2018). Additionally, they also facilitate inter-organism communication between the gut microbiome and the other tissue systems (Nigam 2018). Therefore, adaptive variations in *slc22a21* gene could cumulatively enhance liver detoxification, improve ion reabsorption by the kidneys, and strengthen signaling between the gut microbiome and the central nervous system, all of which would be particularly beneficial during ecological shifts involving changes in salinity and dietary changes as a result of altered nutrient availability. Considering that teleosts occupy diverse niches in different environments, evolution through changes in pleiotropic genes might have been particularly relevant during the initial periods of colonization into new niches.

### How can adaptive variation in convergent genes arise?

In our analysis, the average number of gene copies is generally higher in the convergent orthogroups than in the nonconvergent ones (Fig. 2b). Additionally, some genes show clear differences in the expression of their paralogs (Figures S10 and S12). The presence of multiple copies coupled with the divergence in expression patterns between paralogs could partition the effects of genetic variation, helping overcome any genetic constraint and allowing for variation among paralogs to persist nonadaptively as cryptic divergent variations (hereafter cryptic divergence). Cryptic divergence is the genetic difference between paralogs' variations that have negligible or no phenotypic effects, but during periods of environmental shifts, produce heritable phenotypic variation. It is analogous to cryptic variation among alleles (Paaby and Rockman 2014). The developmental impacts of cryptic variation in pleiotropic genes have been experimentally demonstrated in models like *Drosophila melanogaster*, *C. elegans*, *Arabidopsis thaliana*, and zebrafish (Rutherford and Lindquist 1998; Queitsch et al. 2002; Yeyati et al. 2007; Duveau and Félix 2012). In vivo studies in natural environments, such as those in sticklebacks and cavefish, have highlighted the critical role of ancestral cryptic variation in facilitating the adaptation to ecological changes (McGuigan et al. 2011; Rohner et al. 2013). Additionally, experimental evidence indicates that mutations in protein-coding sequences of enzymes can generate cryptic variation, which maintains the enzyme's native function on its ancestral substrate while enhancing its adaptive potential in a novel substrate (Bloom et al. 2006; Hayden et al. 2011). Therefore, a change in environment can cause the cryptic variation to manifest, leading to phenotypes with enhanced fitness.

Our study has a few caveats. A major requirement for our study was the need for fully annotated chromosomal-level genomes. Most of the teleost fish genomes that fit these criteria were of tropical and subtropical fish species, with comparatively fewer species representing temperate, boreal, and polar fish. Although most species of fish are tropical and subtropical, studying the extent of convergence in other groups would be particularly interesting considering that temperate and boreal fishes have smaller effective population sizes than tropical fish (Daane and Detrich 2022; Bista et al. 2023). Increasing the number of temperate fish would provide insight into how their specific demographic features can influence the degree of convergence. For instance, the convergent patterns we observe may require large effective population sizes where selection as opposed to drift in the primary evolutionary force. As a result, we may observe less convergence in nontropical fishes because of their relatively lower population size and stronger influence of genetic drift, particularly in freshwater fish (Yi and Streelman 2005). Another caveat is the absence of gene expression and genetic perturbation data for the species that exhibit the convergent substitutions, which forced us to use zebrafish as a proxy. It is possible that the convergent genes in these species display a more tissue-specific expression, functioning preferentially in their target tissues as compared to the other tissues, suggesting a coordinated evolution of gene regulation and coding sequences. It would be particularly interesting to explore the possibility of coevolution of mutational effects at the cis-regulatory and coding sequence level (Stern and Orgogozo 2008). This possibility presents an alternative explanation for our findings, which we aim to explore in future studies.

## Materials and methods

### Data collection and estimating orthologs

In our study, we obtained chromosomal-level annotated teleost fish genomes from the NCBI genomes (Sayers et al. 2022) and Ensembl genome databases (Martin et al. 2023) (Table S1). Using an initial list of species, we obtained taxonomic information from FishBase (Sa-a et al. 2000. www.fishbase.org, Houde and Zastrow 1993; Sa-a et al. 2000) and a time-calibrated, rooted species tree from timetree.org (Kumar et al. 2022) (Figure S13). We only included species that were present in the species tree and downloaded their protein and coding sequences. Using the protein sequences from a final set of 143 species, we constructed orthologs using OrthoFinder (Emms and Kelly 2019), resulting in 40,940 orthogroups containing 98.2% of all the genes (Table S5). The final dataset consisted of 9,224 orthogroups with around 2 million orthologous genes (Dataset S1 in online repository).

### Processing of the orthologs

Prior to running the CSUBST algorithm, we executed a series of preprocessing steps, closely following the guidelines outlined in the CSUBST wiki (https://github.com/kfuku52/csubst/wiki/Preparing-CSUBST-inputs). First, we used the CDSKIT v0.91 tool (https://github.com/kfuku52/cdskit) to isolate the longest isoform for each gene and make the sequences in-frame. Next, using TRANSeq v6.6.0 (Madeira et al. 2022), we translated the coding sequences into protein sequences, followed by alignment using MAFFT v7.481 with the “auto” option (Katoh and Standley 2013). We then performed a translation alignment using TRANALIGN v6.6.0 (Madeira et al. 2022) and trimmed poorly aligned sequences using TRIMAL v1.4.1 (Capella-Gutiérrez et al. 2009) with the “*ignorestopcodon*” and “*automated1*” options. Finally, we masked and removed ambiguous sites in the alignment using the “*mask*” and “*hammer*” functions of CDSKIT v0.91.

We used IQ-TREE v2.0.3 (Minh et al. 2020) with the MPF model, and 1,000 ultra-fast optimized bootstrapping replicates (“*bnni*” option), to construct gene trees. We rooted and reconciled the gene trees with the species trees using a combination of GeneRax v1.1.0 (Morel et al. 2020) and NWKIT v0.10.0 (https://github.com/kfuku52/nwkit).

Detailed scripts and commands utilized throughout these steps can be found within the “Scripts” folder in the online repository. We also provide a conda environment YAML file that users can use to recreate the environment for the current preprocessing step as well as the following analysis step. We ran the convergence analysis using a rooted time-calibrated species tree obtained from timetree.org (Kumar et al. 2022).

### Convergence analysis using CSUBST

CSUSBT employs a metric similar to the popular *dN/dS* ratio (parameterized as *ω*), which represents the rate of protein evolution as a ratio between nonsynonymous and synonymous substitution rates (Yang 2014). Distinct from ω, the ω_C_ parameter captures repeated substitutions across various combinations of phylogenetic branches. These combinations range from a minimum of 2 branches, indicating pairwise substitutions, to a maximum of 10 branches. The software quantifies these combinations as *arity (K),* where *K* = 2 represents pairwise comparisons, and *K* = 3 signifies a 3-way branch comparison. By estimating all possible combinations of amino acid substitution (across multiple branch combinations) occurring in a multidimensional sequence space, *ω*_C_ measures how much proteins in different lineages have moved toward the same evolutionary endpoints in this sequence space. CSUBST reports *ω*_C_ statistics for 9 types of combinatorial substitution (Fukushima and Pollock 2023). We focus primarily on omegaCany*2spe*—denoting combinatorial substitution from any ancestral codon to a specific descendant codon, namely, convergent substitutions—and *OCNany2spe*, which denotes the observed rate for nonsynonymous convergence. We ran the CSUSBT algorithm using the “CSUBST *analyze*” command with 5 threads and conducted an exhaustive search for a max *K* = 10. The output of this command can be found in Dataset S4 in the online repository. Orthologs were categorized based on the presence of convergence across these branch combinations and further analyzed using the “*CSUBST site*” command (input files in Dataset S5) to map convergent amino acids onto protein structures. This mapping facilitates the visualization of convergent amino acid sites, particularly their proximity to functional domains within the protein. We employed ad hoc filtering to remove spurious convergence estimates (Figure S2). In this post-processing step, we identified amino acid substitutions that occurred in very close proximity, localized at particular regions of the protein. These are usually caused by misaligned sequences due to splice variants between species. After excluding cases of spurious convergence, we identified 89 orthogroups that showed evidence for reliable convergence. Dataset S6 contains the nonspurious convergence results, while Dataset S7 has examples of spurious convergence.

To detect evidence for purifying selection across the sites we used the Fast Unconstrained Bayesian Approximation (FUBAR) method from the hyphy set of models (Kosakovsky Pond et al. 2020). This Bayesian approach infers synonymous and nonsynonymous substitution rates on a per-site basis for a given coding alignment and phylogeny while being robust to false positives and model misspecification (Murrell et al. 2013).

### Analysis on protein structures and amino acid substitutions

The “*CSUBST site*” command obtains protein structures by searching the RCSB PDB database (RCSB.org) (Berman et al. 2000). If no experimentally determined structures are found, CSUBST uses NCBI's QBLAST (Sayers et al. 2022) search against the UNIPROT database (UniProt Consortium 2023) to obtain a.pdb file from the AlphaFold Protein Structure Database (Varadi et al. 2022). As a result, the structures obtained are often heterogeneous, comprising X-ray crystallography structures of protein in complex with a ligand, noncomplexed protein in crystal, or computationally estimated structures from AlphaFold. This heterogeneity makes statistical comparisons of protein sites difficult. Nonetheless, we applied a systematic selection method to obtain as objective a comparison as possible. We used Pymol (DeLano 2002) version 2.4.5 to systematically classify the residues. For each Pymol session, convergent residues were selected based on their color (red) and surface residues were determined using a cutoff of 1.4 Å^2^ to determine their exposure to the solvent. Residues with a distance less than or equal to 4 Å from the ligand were classified as in the vicinity of the ligand. Buried residues were those that did not have access to the surface or internal cavity of the protein. The secondary structures of helices, sheets, and loops are selected directly using the keywords provided in Pymol. For all these selections, a second b-factor parameter > 0 was added to eliminate residues that were present in the sequence but not built into the structure. From this set, we can then cross-reference the selections to determine the desired list and number of residues. These results are in Table S7. Using the *CSUBST site* output files (Dataset S6), we obtained the ancestral and extant amino acids for both convergent and nonconvergent substitutions. We compared the average BLOSUM62 scores between the 2 types of substitutions using the pwalign R package (Aboyoun and Gentleman 2024). We obtained data for the physicochemical properties of amino acid residues from the Peptide R package (Osorio et al. 2015). For each protein, we obtained the values of the ancestral amino acid properties for the 2 types of substitutions and tested for significant changes from the ancestral condition to the extant convergent condition, and filtered out the number of significant changes (Fig. 2d). Figures showing the difference in properties for each protein can be found in Dataset S11.

### Analysis of GO terms

We used DeepGOPlus v1.0.15 (Kulmanov and Hoehndorf 2020) to annotate all the genes (∼4 million) in our dataset with gene ontology (GO) terms. DeepGOPlus integrates a convoluted neural network along with sequence similarity scores of protein sequences to annotate GO terms. In our analysis, we use a prediction threshold of 0.3 (Kulmanov and Hoehndorf 2020). Using the GOstats and GO.db Bioconductor (Falcon and Gentleman 2007) packages, we performed GO enrichment for GO terms related to biological processes. In this analysis, convergent orthologs constituted the test set, while the remainder served as the background universe. Enriched terms were identified using a Benjamini–Hochberg corrected *P* cutoff of <0.05. For the semantic similarity analysis, we used GO annotation of *D. rerio* orthologs obtained from GO.db (Falcon and Gentleman 2007) and carried out the analysis using the GOSemSim R package (Yu 2020). We computed pairwise semantic similarity between GO terms for 2 gene sets; the convergent *D. rerio* orthologs and a random set of orthologs used as background. We used multiple random sets and always obtained the same results. We used the *Wang* measure of semantic similarity using *BMA* aggregation (Yu 2020).

### Testing for differences between distributions

We used a bootstrapped version of the Kolmogorov–Smirnov (KS) test when comparing the distributions of features between convergent and nonconvergent genes. The KS type tests are nonparametric distance tests used to compare distributions. However, there can be instances (e.g. unbalanced samples or heavy-tailed data) where determining the asymptotic distributions of the test statistics under the null hypothesis is not possible due to violations of the underlying assumptions of the distribution of the data. Therefore, we used the bootstrap approach described in (Abadie 2002) to build an empirical null distribution that captures the spread of the data based on the actual empirical distributions of the data, rather than using the empirical cumulative distribution function from the asymptotic prediction. It comprises the following steps:

1. Compute
  $$T_{\mathrm{obs}}={\left(\frac{n_{1}n_{0}}{n}\right)}^{1/2}\sup{\;}_{\left\{y\in R\right\}}|F_{1,n_{1}}(y)-F_{0,n_{0}}(y)|$$

The KS-type test statistic from the observed data, where $F_{0,n_{0}}(y)$ is the data (with $n_{0}$ observations) corresponding to the sample variable being equal to 0 and $F_{1,n1}(y)$ is the data (with *n*_1_ observations) corresponding to the sample variable being equal to 1. Here 0 and 1 could indicate background genes and convergent genes.

2. Resample $n_{\;}$ observations with replacement from the pooled data and divide this into 2 sets, first set comprising the first $n_{1}$ observations and second of $n_{0}$ observations. Use these 2 sets of observations to re-compute the test statistic $T_{obs}$ to $T_{obs},i.$
3. Repeat step 2 *N* times. In our case we run 5,000 bootstraps.
4. Compare $T_{obs}\;$ to $T_{obs,i}\;$ with the *P*-value being the proportion of bootstrap test statistics more extreme than the observed one:
  $$\frac{1}{N}\overset{N}{\underset{i=1}{\sum }}\;I(\hat{T_{obs,i}\;}>\;T_{obs})$$

where $I(\hat{T_{obs,i}\;}>\;T_{obs})$ is an indicator variable that equals 1 when $T_{obs,i}\;$ > $T_{obs}\;$ and 0 otherwise. This is the probability of obtaining a distance $T_{obs}\;$ under the null hypothesis. We used the R package ksboot to implement this test. (https://rdrr.io/cran/kldtools/man/ksboot.html).

### Bgee data and estimating tau

To gain deeper insight into the functional relevance of the convergent genes, we used data from the Bgee database (Bastian et al. 2025) and studied expression across 11 species (*Astyanax mexicanus*, *A. calliptera*, *D. rerio*, *Esox lucius*, *Gasterosteus aculeatus*, *Gadus morhua*, *Neolamprologus brichardi*, *Nothobranchius furzeri*, *Oryzias latipes*, *S. salar*, and *Scophthalmus maximus*) and 8 tissues. We calculated tissue specificity for the brain, eye, heart, liver, muscle tissue, ovary, and testis since they were the tissues with the most consistent sampling across species. All species had gene expression data for these tissues. To calculate tissue specificity, we used the tau metric because of its efficiency in recovering biological signals (Kryuchkova-Mostacci and Robinson-Rechavi 2016). We classified a gene as tissue-specific if it had a tau ≥ 0.8 and its expression in the target tissue was greater than the sum of its expression in other tissues. Since we are comparing tissue specificity across different species, we identified genes that are tissue-specific in the same tissue across all the species sampled. These criteria ensured we captured robust signals for tissue specificity. We also use data from Bgee to validate functional expression of genes in species harboring the convergent substitutions. To enable robust comparisons across conditions, experiments, and data types, we use the expression scores computed by Bgee (Bastian et al. 2025). These scores provide a normalized measure of a gene's expression across multiple anatomical and developmental conditions (Bastian et al. 2025). In this framework, higher expression scores indicate stronger expression in a given anatomical entity and developmental stage. This approach offers a consistent and quantitative assessment of the spatial and temporal breadth of gene expression within each species.

Using this metric, we show that the convergent genes have a wide spatial and temporal breadth, being expressed across multiple tissues and different developmental stages (Figure S10).

### Analysis of single-cell RNA-Seq from zebrahub

We used single-cell RNA-seq (scRNAseq) from the Zebrahub consortium (Lange et al. 2024). These data comprise a high-resolution scRNAseq atlas of zebrafish development achieved by sequencing individual embryos across 10 developmental stages (Lange et al. 2024). The dataset contained 120,444 cells and a total of 529 cell clusters, which were annotated based on published literature and the ZFIN database (Bradford et al. 2022). The entire dataset is available at https://zebrahub.ds.czbiohub.org/. The consortium provides h5ad objects with quality control, clustering, and uniform manifold projections (UMAP, which are a dimensionally reduced representation of high-dimensional data) (https://github.com/czbiohub-sf/zebrahub_analysis). We log-normalized this processed data using the scater R package (McCarthy et al. 2017) and used it for our study. To visualize the expression of the convergent genes across embryonic development, we calculated the mean expression of each gene at each time point and constructed a scaled (max scaled to 1) heatmap using the scuttle (McCarthy et al. 2017) and dittoSeq (Bunis et al. 2021) R packages. We visualized heatmaps using the plotReducedDim function from the scater R package (McCarthy et al. 2017). All UMAP plots with cell type and gene expression at each time point are available in Dataset S9. To estimate distribution of gene expression in cell types, we first filtered out low-expressed genes (log counts < 0.5) and then selected cells that had expression across all the sampled fish for each timepoint. This was done to ensure we obtain a consistent gene expression profile across timepoints. We then calculated the number of unique cell types each gene was expressed in. Using this data, we estimated the distribution of cell type expression for the convergent and nonconvergent gene sets.

## Supplementary Material

msag015_Supplementary_Data

## Data Availability

All data comprising sequences, output files, figures, code, and datasets are available in the online repository: https://doi.org/10.5281/zenodo.17921689. An electronic report containing output of R code and major analyses in this study can be found at: https://agneeshbarua.github.io/Teleost_convergence/
