# Supplementary material for: Estimates of molecular convergence reveal multiple genes with adaptive variation across teleost fish
Source: Mol Biol Evol. 2026 Jan 28;43(1):msag015. doi: 10.1093/molbev/msag015 (PMC12849823; doi:10.1093/molbev/msag015)
Supplement: msag015_Supplementary_Data [file msag015_supplementary_data.zip › MBE-25-0803.R3_SUPP.pdf]

Supplementary material for: Estimates of molecular convergence reveal multiple genes with adaptive variation across teleost fish

Agneesh Barua[1,2*], Malvika Srivastava[1], Brice Beinsteiner[3], Vincent Laudet[4,5], Marc Robinson-Rechavi[1,2]

[1]Department of Ecology and Evolution, University of Lausanne

[2]Swiss Institute of Bioinformatics

[3]Helmholtz Pioneer Campus, Helmholtz Zentrum München, Neuherberg, Germany

[4]Marine Eco-Evo-Devo Unit, Okinawa Institute of Science and Technology Graduate University, Japan

[5]Marine Research Station, Institute of Cellular and Organismic Biology (ICOB), Academia Sinica, 23-10, Dah-Uen Rd, Jiau Shi, I-Lan 262, Taiwan

An electronic report containing output of R code used in this study can be found at:

https://agneeshbarua.github.io/Teleost_convergence/

All data comprising sequencers, output files, figures, and code are available in the Zenodo data repository: https://doi.org/10.5281/zenodo.17631061

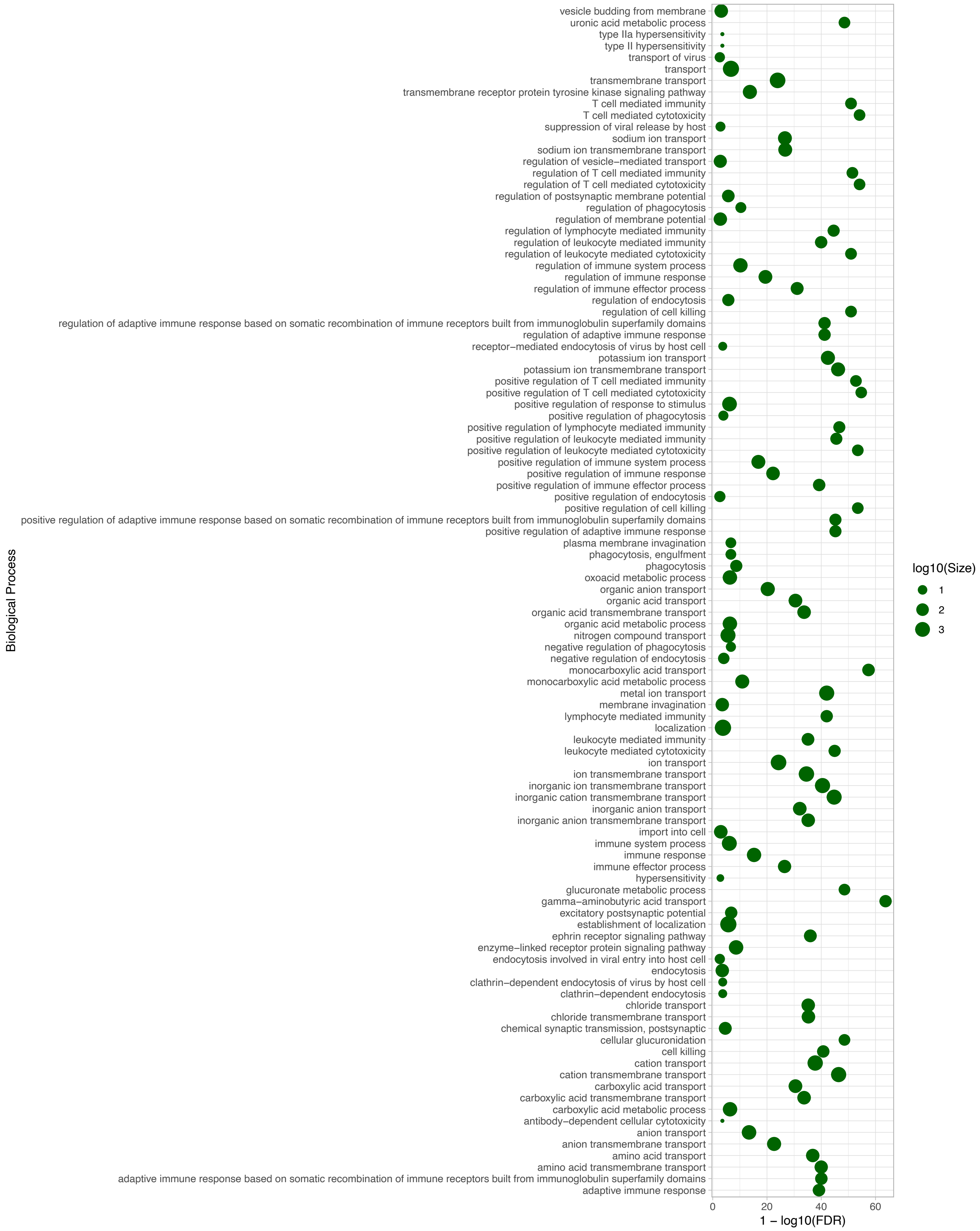
vesicle budding from membrane
uronic acid metabolic process
type IIa hypersensitivity
type II hypersensitivity
transport of virus
transport
transmembrane transport
transmembrane receptor protein tyrosine kinase signaling pathway
T cell mediated immunity
T cell mediated cytotoxicity
suppression of viral release by host
sodium ion transport
sodium ion transmembrane transport
regulation of vesicle−mediated transport
regulation of T cell mediated immunity
regulation of T cell mediated cytotoxicity
regulation of postsynaptic membrane potential
regulation of phagocytosis
regulation of membrane potential
regulation of lymphocyte mediated immunity
regulation of leukocyte mediated immunity
regulation of leukocyte mediated cytotoxicity
regulation of immune system process
regulation of immune response
regulation of immune effector process
regulation of endocytosis
regulation of cell killing
regulation of adaptive immune response based on somatic recombination of immune receptors built from immunoglobulin superfamily domains
regulation of adaptive immune response
receptor−mediated endocytosis of virus by host cell
potassium ion transport
potassium ion transmembrane transport
positive regulation of T cell mediated immunity
positive regulation of T cell mediated cytotoxicity
positive regulation of response to stimulus
positive regulation of phagocytosis
positive regulation of lymphocyte mediated immunity
positive regulation of leukocyte mediated immunity
positive regulation of leukocyte mediated cytotoxicity
positive regulation of immune system process
positive regulation of immune response
positive regulation of immune effector process
positive regulation of endocytosis
positive regulation of cell killing
positive regulation of adaptive immune response based on somatic recombination of immune receptors built from immunoglobulin superfamily domains
positive regulation of adaptive immune response
plasma membrane invagination
phagocytosis, engulfment
phagocytosis
oxoacid metabolic process
organic anion transport
organic acid transport
organic acid transmembrane transport
organic acid metabolic process
nitrogen compound transport
negative regulation of phagocytosis
negative regulation of endocytosis
monocarboxylic acid transport
monocarboxylic acid metabolic process
metal ion transport
membrane invagination
lymphocyte mediated immunity
localization
leukocyte mediated immunity
leukocyte mediated cytotoxicity
ion transport
ion transmembrane transport
inorganic ion transmembrane transport
inorganic cation transmembrane transport
inorganic anion transport
inorganic anion transmembrane transport
import into cell
immune system process
immune response
immune effector process
hypersensitivity
glucuronate metabolic process
gamma−aminobutyric acid transport
excitatory postsynaptic potential
establishment of localization
ephrin receptor signaling pathway
enzyme−linked receptor protein signaling pathway
endocytosis involved in viral entry into host cell
endocytosis
clathrin−dependent endocytosis of virus by host cell
clathrin−dependent endocytosis
chloride transport
chloride transmembrane transport
chemical synaptic transmission, postsynaptic
cellular glucuronidation
cell killing
cation transport
cation transmembrane transport
carboxylic acid transport
carboxylic acid transmembrane transport
carboxylic acid metabolic process
antibody−dependent cellular cytotoxicity
anion transport
anion transmembrane transport
amino acid transport
amino acid transmembrane transport
adaptive immune response based on somatic recombination of immune receptors built from immunoglobulin superfamily domains
adaptive immune response
Biological Process
0
20
40
60
1 − log10(FDR)
log10(Size)
1
2
3

Fig S1: Gene Ontology (GO) term enrichment of excluded orthogroups. To keep computational times reasonable we restricted our analysis to orthogroups containing a maximum of 1500 genes. We annotated and functionally characterised the excluded orthogroups and found that they were mostly associated with immune processes, metabolisms, and cell signalling. Although processes related to cell signalling and immunity have substantial functional significance in organisms, our sampled gene sets encompassed a more diverse array of processes (Fig S6).

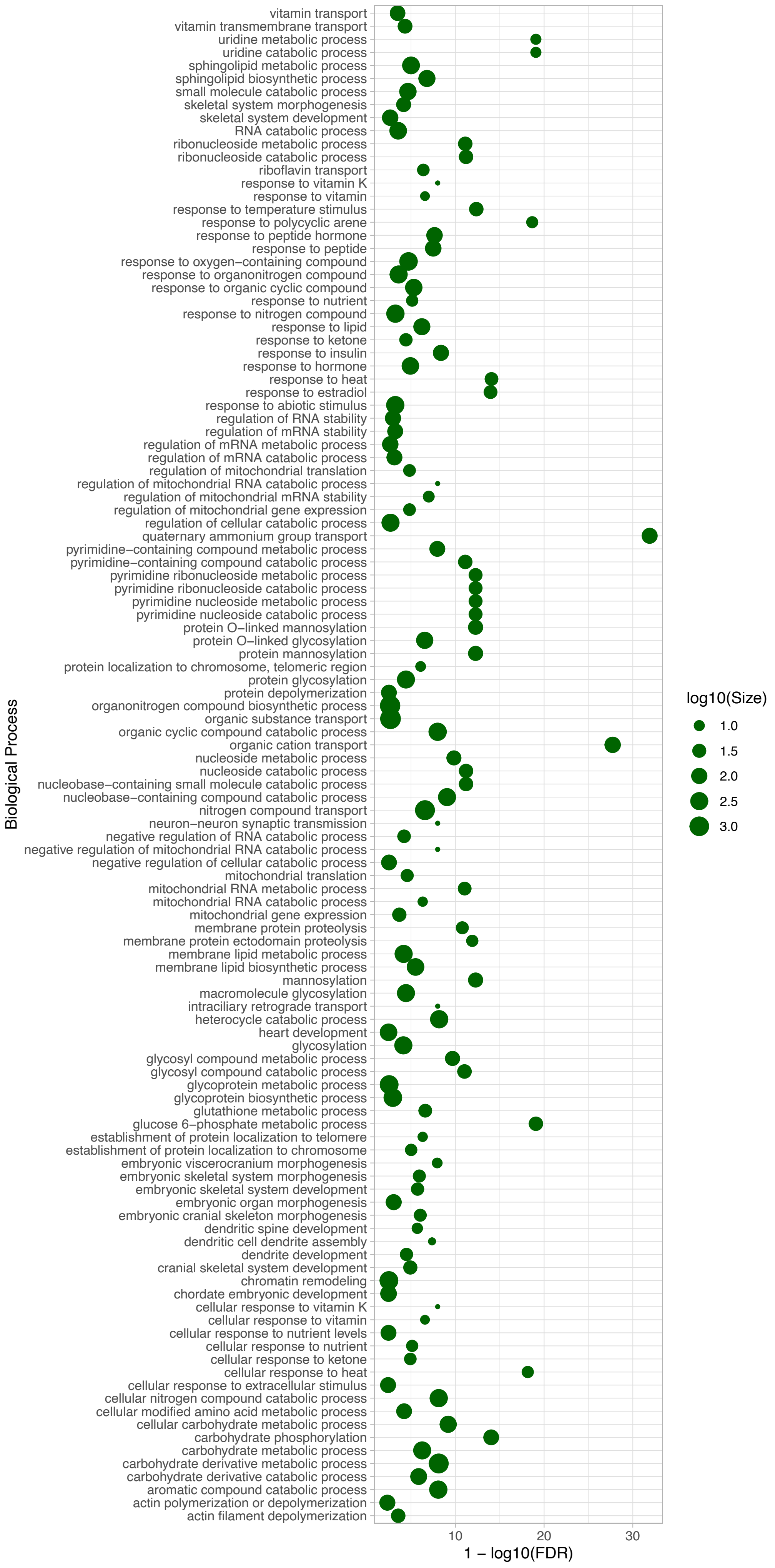
vitamin transport
vitamin transmembrane transport
uridine metabolic process
uridine catabolic process
sphingolipid metabolic process
sphingolipid biosynthetic process
small molecule catabolic process
skeletal system morphogenesis
skeletal system development
RNA catabolic process
ribonucleoside metabolic process
ribonucleoside catabolic process
riboflavin transport
response to vitamin K
response to vitamin
response to temperature stimulus
response to polycyclic arene
response to peptide hormone
response to peptide
response to oxygen−containing compound
response to organonitrogen compound
response to organic cyclic compound
response to nutrient
response to nitrogen compound
response to lipid
response to ketone
response to insulin
response to hormone
response to heat
response to estradiol
response to abiotic stimulus
regulation of RNA stability
regulation of mRNA stability
regulation of mRNA metabolic process
regulation of mRNA catabolic process
regulation of mitochondrial translation
regulation of mitochondrial RNA catabolic process
regulation of mitochondrial mRNA stability
regulation of mitochondrial gene expression
regulation of cellular catabolic process
quaternary ammonium group transport
pyrimidine−containing compound metabolic process
pyrimidine−containing compound catabolic process
pyrimidine ribonucleoside metabolic process
pyrimidine ribonucleoside catabolic process
pyrimidine nucleoside metabolic process
pyrimidine nucleoside catabolic process
protein O−linked mannosylation
protein O−linked glycosylation
protein mannosylation
protein localization to chromosome, telomeric region
protein glycosylation
protein depolymerization
organonitrogen compound biosynthetic process
organic substance transport
organic cyclic compound catabolic process
organic cation transport
nucleoside metabolic process
nucleoside catabolic process
nucleobase−containing small molecule catabolic process
nucleobase−containing compound catabolic process
nitrogen compound transport
neuron−neuron synaptic transmission
negative regulation of RNA catabolic process
negative regulation of mitochondrial RNA catabolic process
negative regulation of cellular catabolic process
mitochondrial translation
mitochondrial RNA metabolic process
mitochondrial RNA catabolic process
mitochondrial gene expression
membrane protein proteolysis
membrane protein ectodomain proteolysis
membrane lipid metabolic process
membrane lipid biosynthetic process
mannosylation
macromolecule glycosylation
intraciliary retrograde transport
heterocycle catabolic process
heart development
glycosylation
glycosyl compound metabolic process
glycosyl compound catabolic process
glycoprotein metabolic process
glycoprotein biosynthetic process
glutathione metabolic process
glucose 6−phosphate metabolic process
establishment of protein localization to telomere
establishment of protein localization to chromosome
embryonic viscerocranium morphogenesis
embryonic skeletal system morphogenesis
embryonic skeletal system development
embryonic organ morphogenesis
embryonic cranial skeleton morphogenesis
dendritic spine development
dendritic cell dendrite assembly
dendrite development
cranial skeletal system development
chromatin remodeling
chordate embryonic development
cellular response to vitamin K
cellular response to vitamin
cellular response to nutrient levels
cellular response to nutrient
cellular response to ketone
cellular response to heat
cellular response to extracellular stimulus
cellular nitrogen compound catabolic process
cellular modified amino acid metabolic process
cellular carbohydrate metabolic process
carbohydrate phosphorylation
carbohydrate metabolic process
carbohydrate derivative metabolic process
carbohydrate derivative catabolic process
aromatic compound catabolic process
actin polymerization or depolymerization
actin filament depolymerization
Biological Process
10
20
30
1 − log10(FDR)
log10(Size)
1.0
1.5
2.0
2.5
3.0

Fig S2: GO term enrichment of the convergent substitutions. GO term enrichment of the convergent genes showed that they were inolved in processes related to biomolecule metabolism, response to hormones or stimuli such as heat, and processes of embryonic development and tissue morphogenesis. This underscores the potential multifunctional nature of these convergent genes.

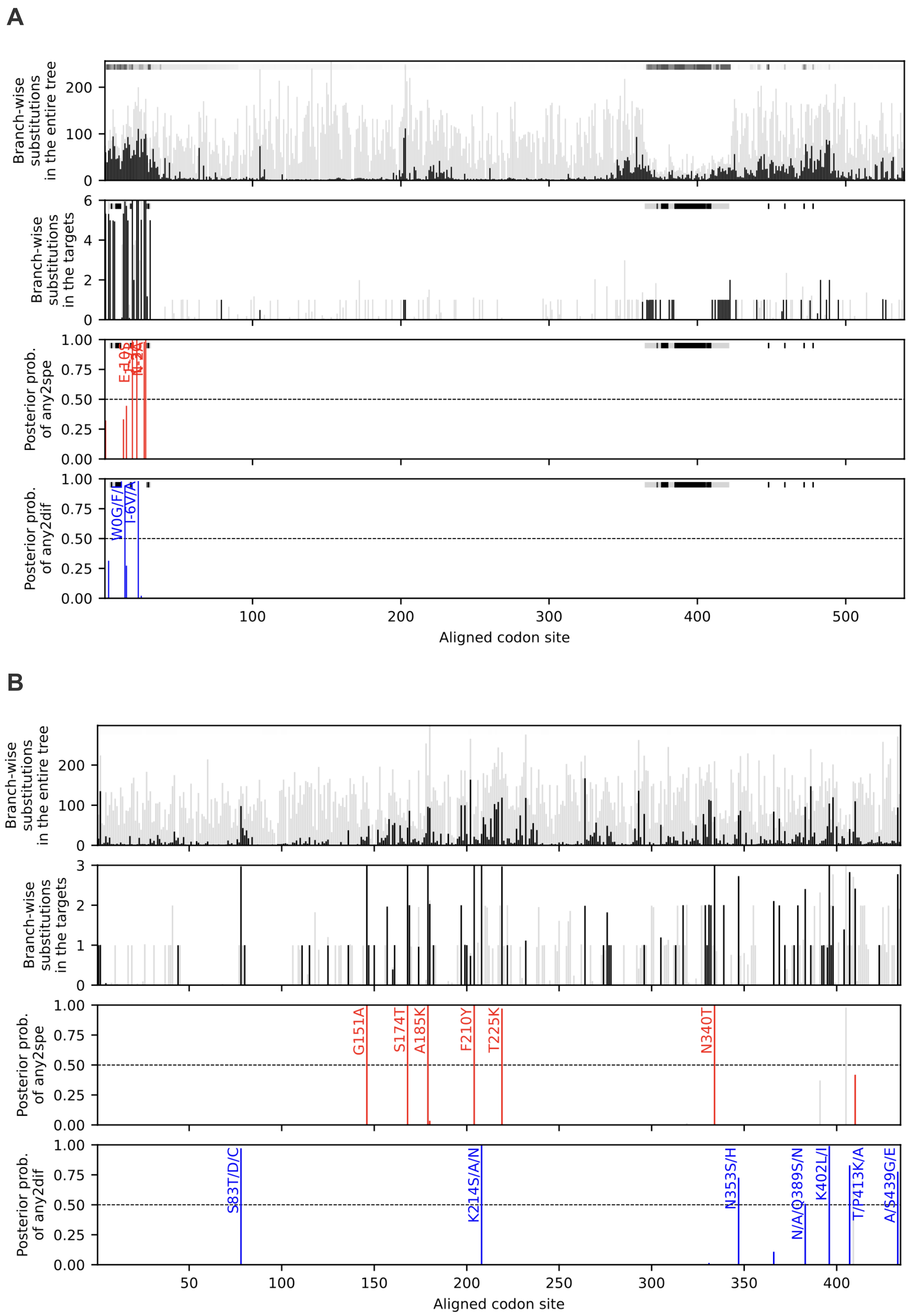

A
Branch-wise substitutions in the entire tree
0
100
200
Branch-wise substitutions in the targets
0
2
4
6
Posterior prob. of any2spe
0.00
0.25
0.50
0.75
1.00
Posterior prob. of any2dif
W0G/F/
I-6V/A
100
200
300
400
500
Aligned codon site
B
Branch-wise substitutions in the entire tree
Branch-wise substitutions in the targets
0
1
2
3
Posterior prob. of any2spe
G151A
S174T
A185K
F210Y
T225K
N340T
Posterior prob. of any2dif
S83T/D/C
K214S/A/N
N353S/H
N/A/Q389S/N
K402L/I
T/P413K/A
A/S439G/E
50
150
250
350
Aligned codon site

Fig S3: Check for spurious convergence: There can be certain instances of spurious convergence events even when stringent convergence metrics are used. This can often occur due to misalignment of sequences, which might be due to splice variants represented inconsistently among different species. Fukushima and Pollock also encountered this in their original publication when analysing human and mouse sequences (Supplementary text 12 in (1). This suggests that spurious convergence events are not restricted to any specific dataset but is an artefact that has to be corrected. (A) The characteristic of these spurious convergence events is their unnatural localisation on the protein structures that can be detected in the output of the CSUBST site function. A workaround would be to select convergence events that are not located at proximity to one another. We perform this ad-hoc filtering by selecting orthologs where the individual substitutions have a high OCN value and are well separated on the protein structure. (B) This helps us identify reliable patterns of convergence as shown. In the above plots black and grey vertical bars represent non-synonymous and synonymous substitutions repsectively. The black horizontal bars in panel A represent gaps in mapping the alignment to the protein structure. The posterior probability of *any2spe* represents the site-wise posterior probabilities of a substitution from a differnt acestral amino acid to a specific extant amino acid, i.e. convergent substitutions, while *any2diff* represents a substitution from any ancestral amino acid to a different extant amino acid. These demarkations were used in Fig 2 of the main text.

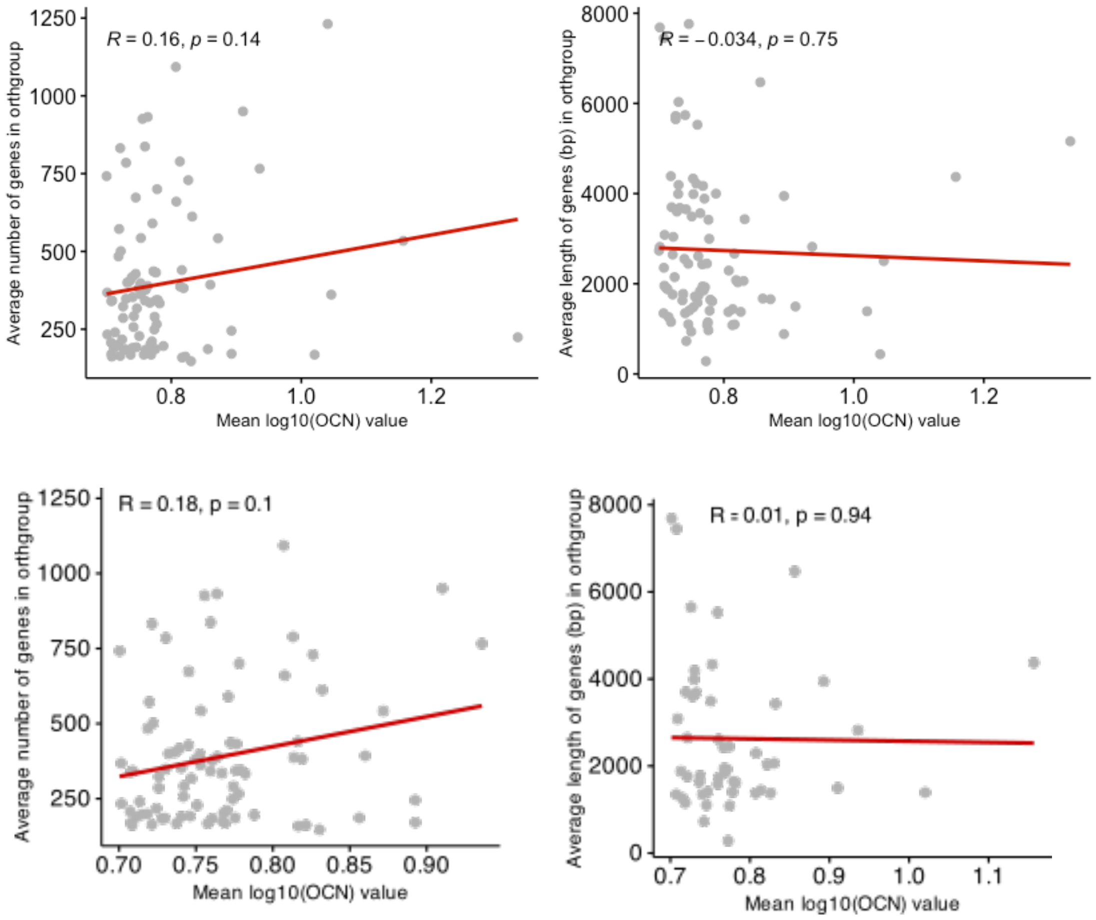

Fig S4: Observed rate of convergence (OCN) versus orthogroups' characterisitcs. We checked for the presense of any potential bias in between OCN values and the average length and number of genes in each orthogroup (top panel). Using a Pearson's correlation test we found no significant relationship between gene number or gene length and the OCN metric, suggesting that the OCN value strictly depends on sequence variation, and that such bias is not a concern in our data. We removed outliers with high OCN values and observed the same lack of correlation (bottom panel).

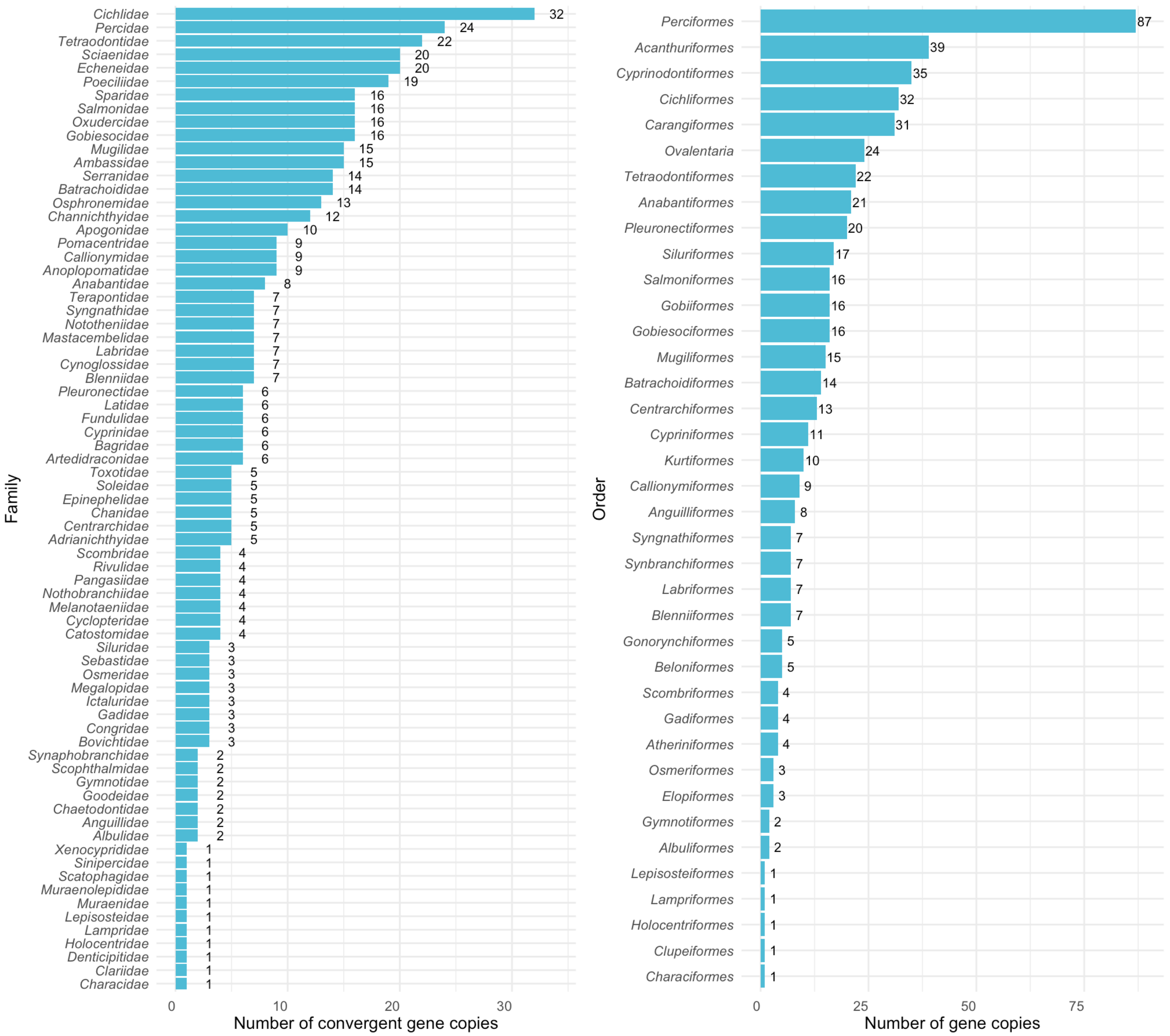

Continued on next page

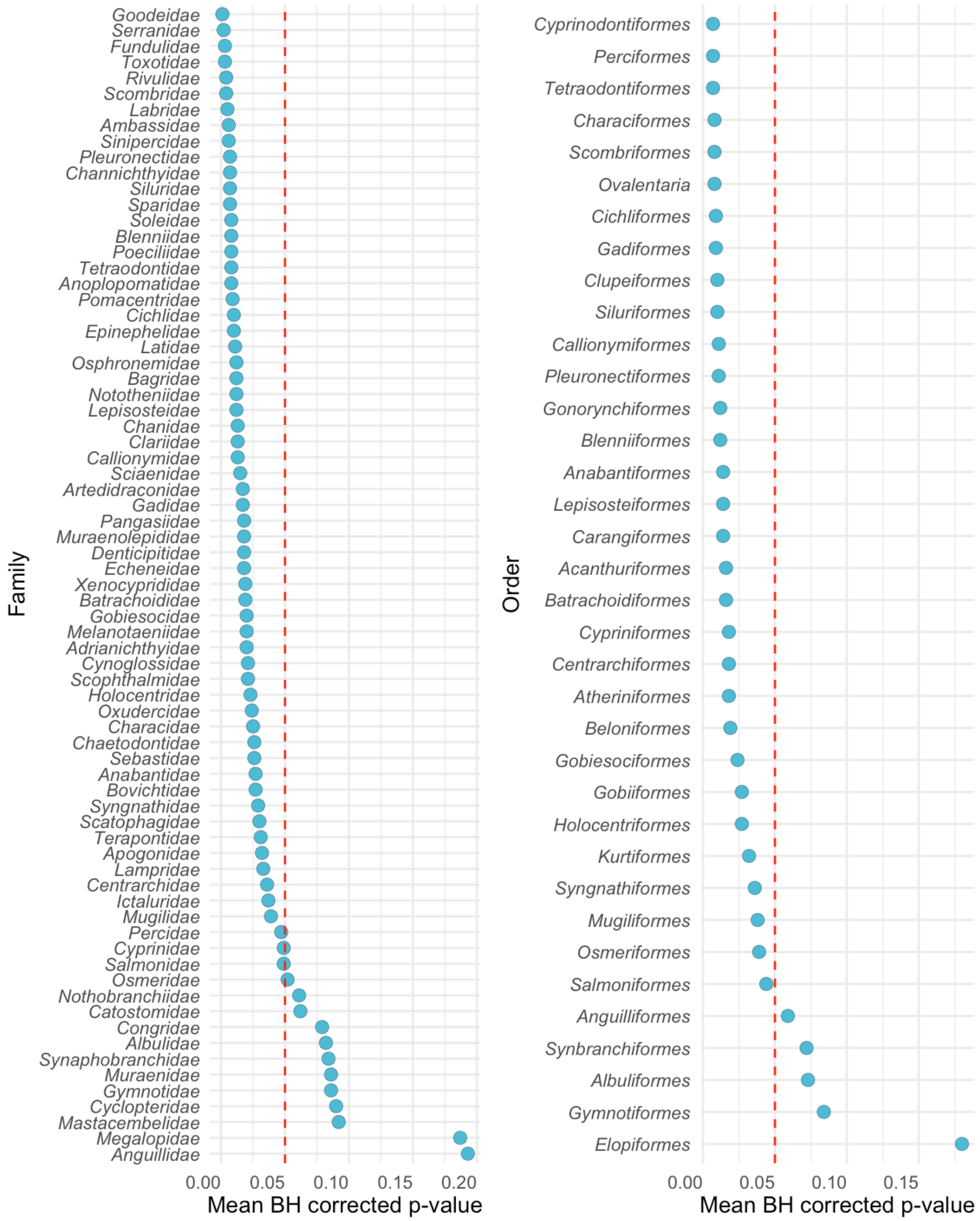

Fig S5: Copy number distribution of the convergent orthogroups. Top panel shows the number of gene copies present in the convergent orthogroups across teleost family and order. Bottom panel shows the mean Benjamini-Hochberg corrected P values for a one sided KS test to determine difference in gene copy number distributions between convergent orthogroups and background sets. Beyond the global trend of high copy number in the convergent orthogroups, we also observed similar trends within individual clades. At the order level, 86% (31 out of 36) of clades showed evidence of higher copy numbers among convergent genes, and at the family level, 83% (61 out of

73) showed the same pattern. Thus, while the majority of clades had higher copy numbers in convergent orthogroups, this trend was not universal across all teleost lineages

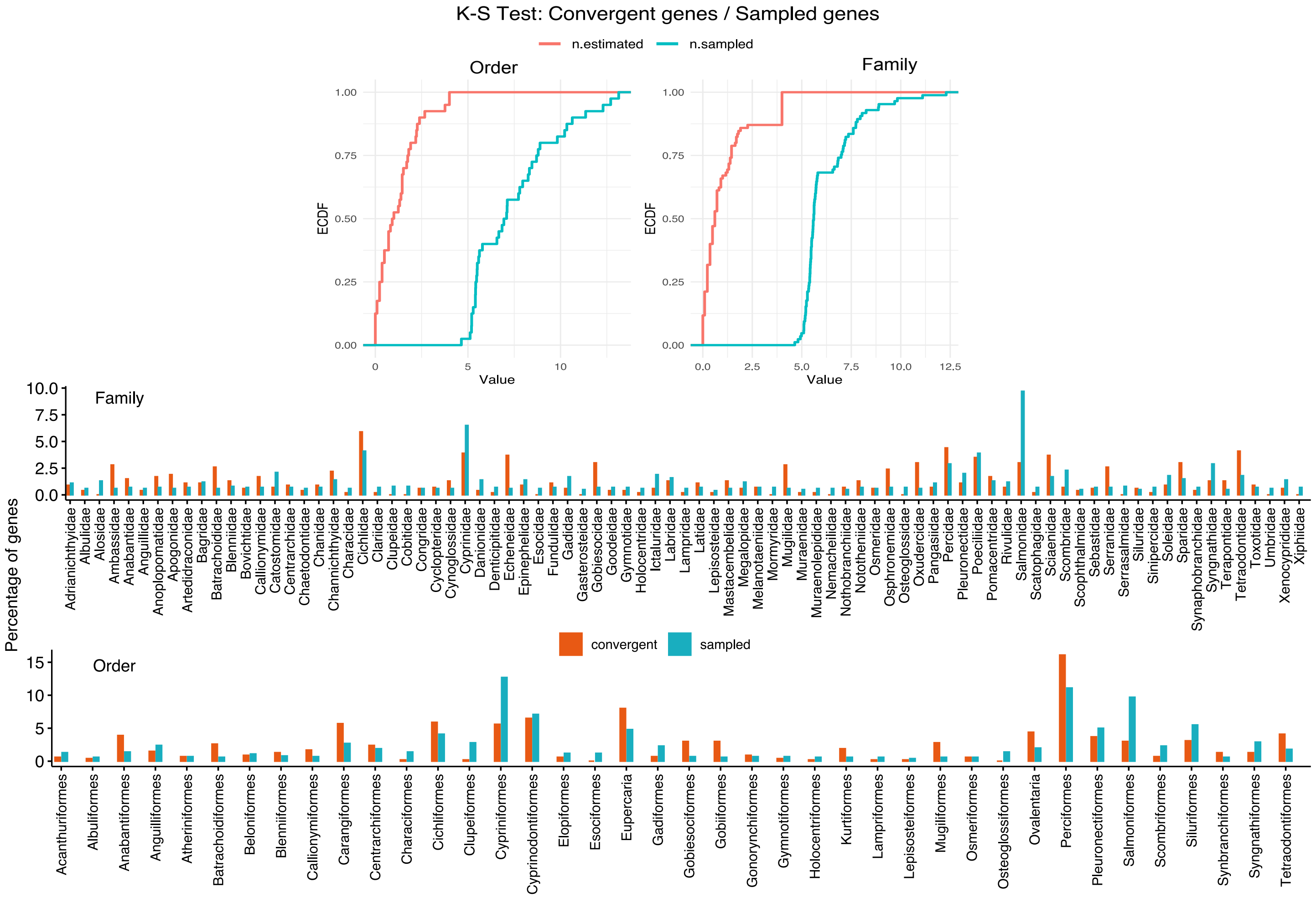

Fig S6: Comparing the distribution of convergent orthogroups versus sampled genes from each order. We use the Kolmogorov-Smirnov test (KS test) to compare distributions of total number of genes sampled versus number of convergent genes estimated (top panel). The rationale is to check whether the relative distributions of the convergent genes are different from that of the number of genes that were sampled. This will tell us whether the deviation between the number of gene sampled in each order/family and the number of genes found convergent is meaningful. The KS test is done with bootstrapping which is suitable for frequency distributions of discrete variables (number of genes in each order). See https://rdrr.io/cran/kldtools/man/ksboot.html. Observe that the shape of the empirical cumulative probability distributions (ECDF) are different for the convergent genes (red) and total sampled genes (blue). The differences in frequencies is further visualised in the ar plots.

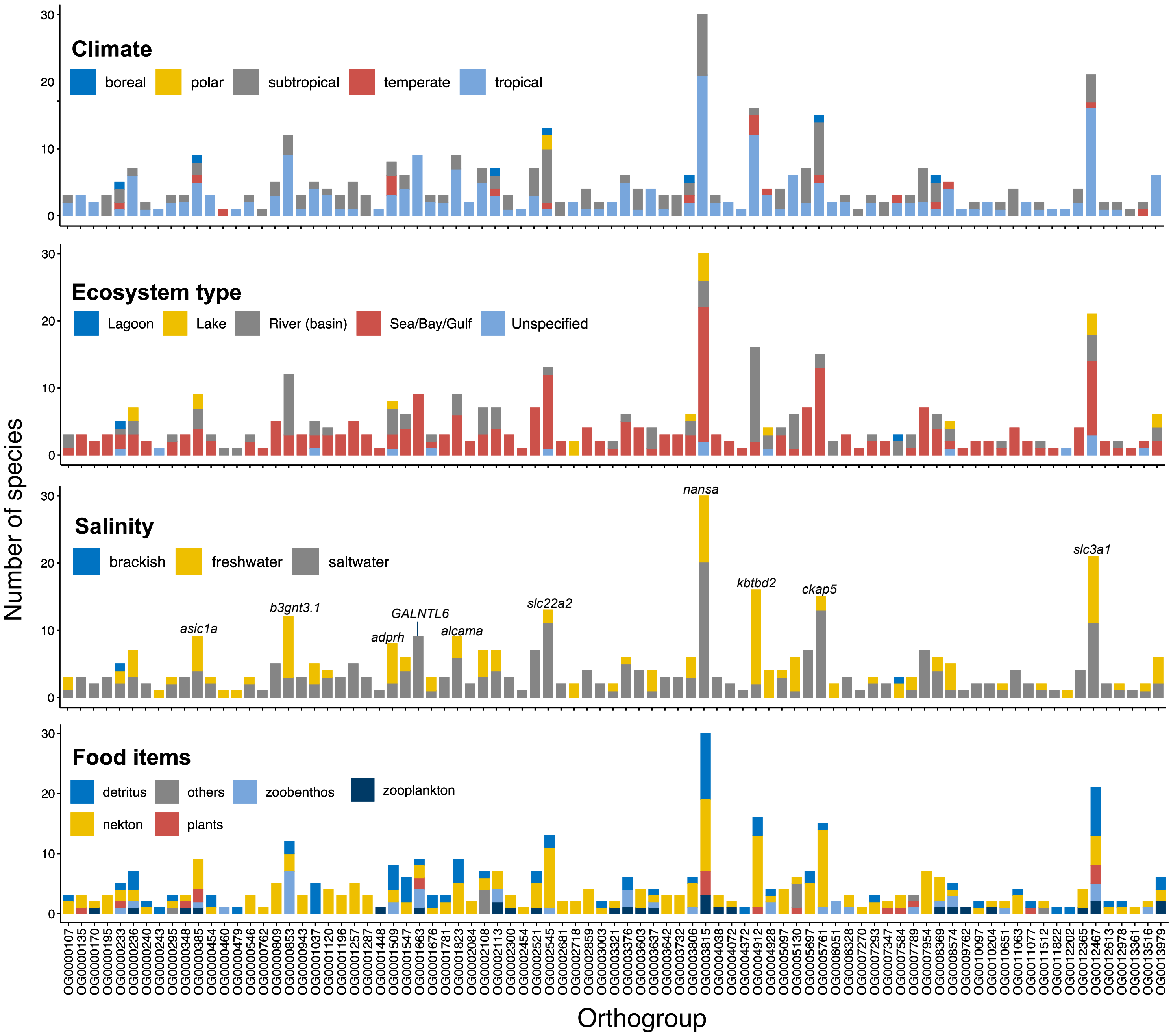

Fig S7: Ecological characteristics of species with convergent genes. The bar plots show ecological characteristics of species that harbour convergent substitutions. The data were obtained from the FishBase database. The proportions are based on species with available data. The gene names or orthogroups with a high number of species are labelled. We performed tests of independence to infer whether there is any relationship between convergence in one orthogroup and the ecological characters. After correcting for multiple testing, only 4 orthogroups show a significant relationship. However, these relationships are with the undefined food variable 'other'. As a result, we cannot make any biological relevant inference from this relationship.

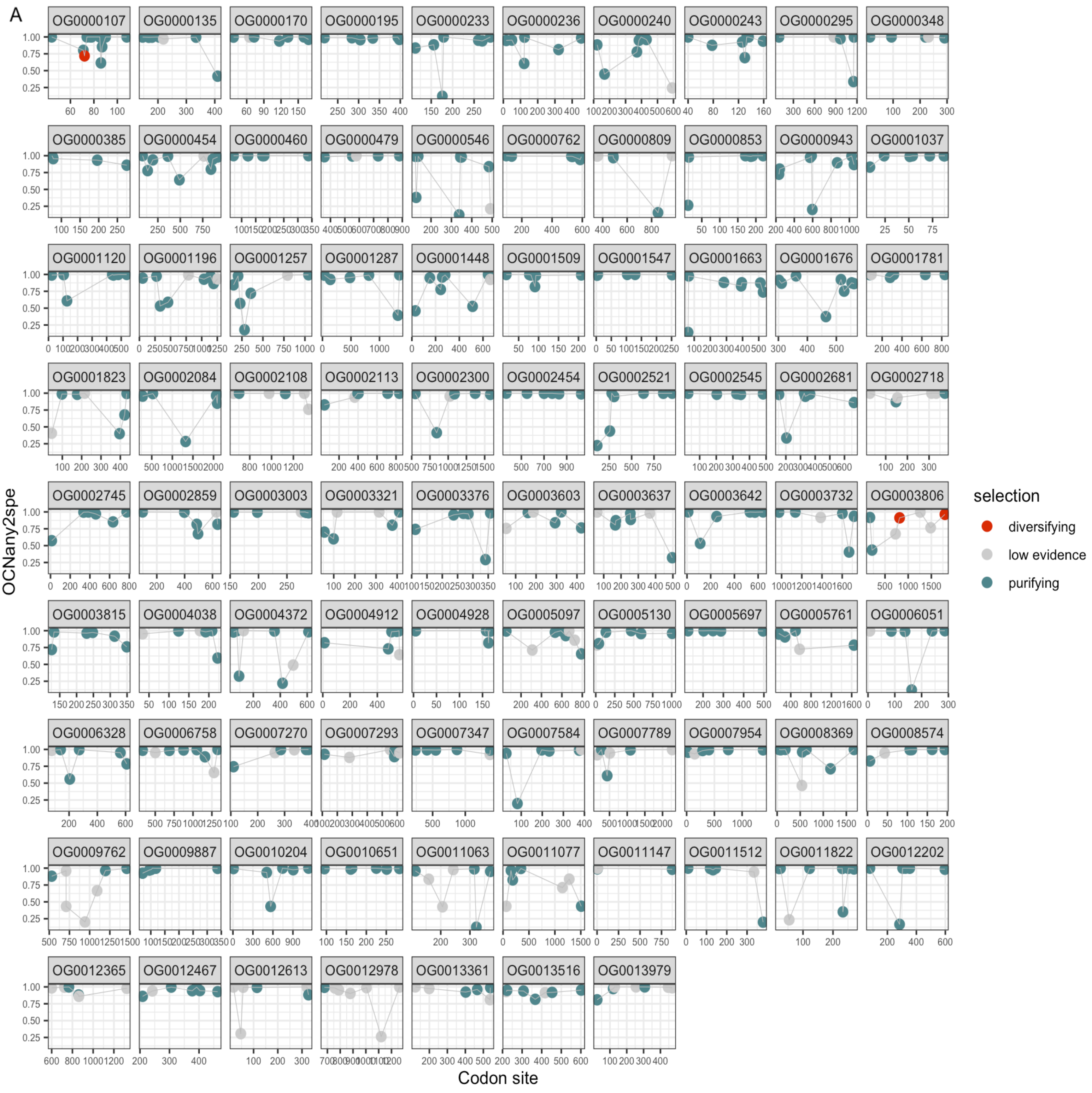

Continued on next page

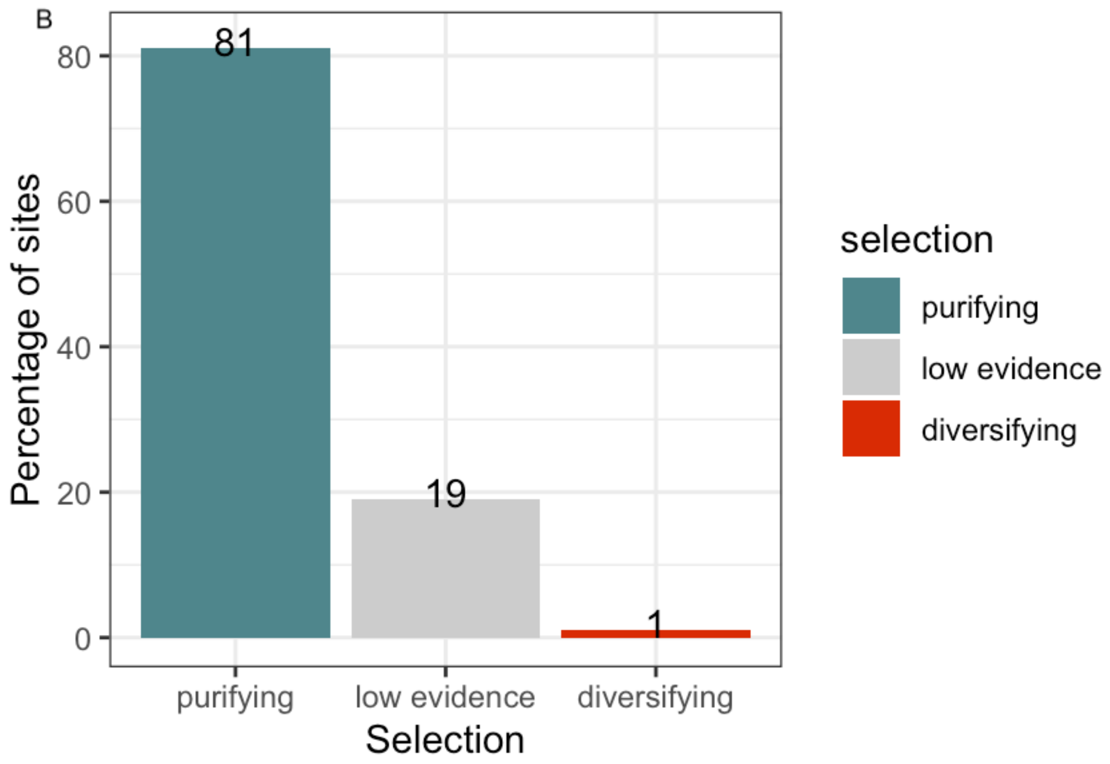

Fig S8 Fast Unconstrained Bayesian Approximation (FUBAR) of selective regime. Using FUBAR we estimated selective regime at each convergent site across the teleosts. (A) The $OCN_{any\text{-}to\text{-}specific}$ convergent metric value and the selective regime experienced by that site. This figure shows all non-zero $OCN_{any\text{-}to\text{-}specific}$ values. In our analyses we only considered sites with an $OCN_{any\text{-}to\text{-}specific}$ above 0.5. (B) The proprtion of convegent sites and their underlying selective regimes.

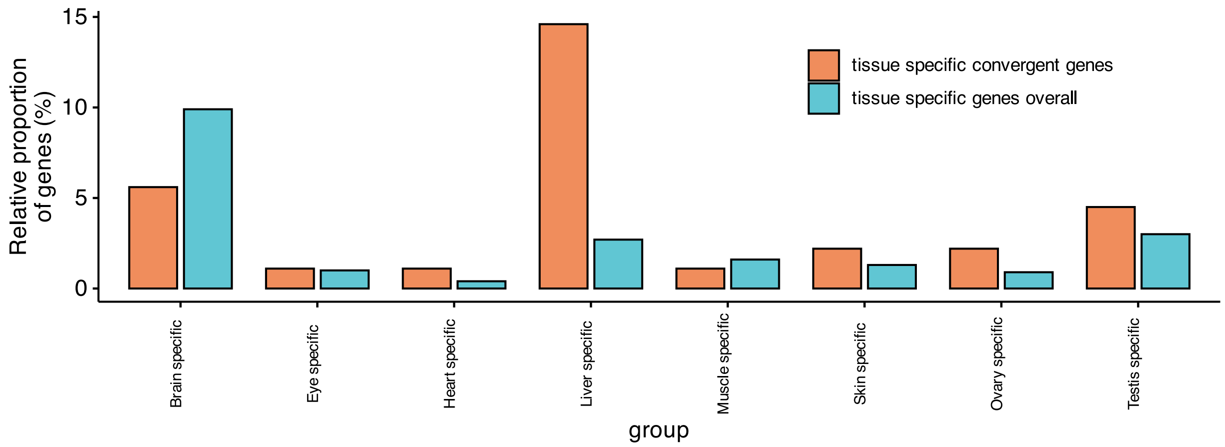

Fig S9: The proportion of tissue specific genes in convergent genes and genes overall for different tissue types**:** We collected gene expression data for the brain, eye, heart, liver, muscle tissue, ovary, and testis across eleven species (*Astyanax mexicanus, Astatotilapia calliptera, Danio rerio, Esox lucius, Gasterosteus aculeatus, Gadus morhua, Neolamprologus brichardi, Nothobranchius furzeri, Oryzias latipes, Salmo salar,* and *Scophthalmus maximus*). We classified a gene as tissue-specific if it had a tau > 0.8 and its expression in the target tissue was greater than the sum of its expression in other tissues. Since we are comparing tissue specificity across different species, we identified genes that are tissue-specific in the same tissue across all the species sampled. These criteria ensured we captured robust signals for tissue specificity. Around one-third of these genes had signals of convergent evolution with the largest being in the liver. We observed that the proportion of convergent genes that are tissue specific is no different from the total proportion of tissue specific genes in our dataset. However, using Fisher's exact test we found a significant difference in the convergent/non-convergent ratio between tissue-specific and non-tissue-specific genes only for the liver and not for other tissues; in other words, our convergent gene set had a higher proportion of liver-specific genes than expected by chance.

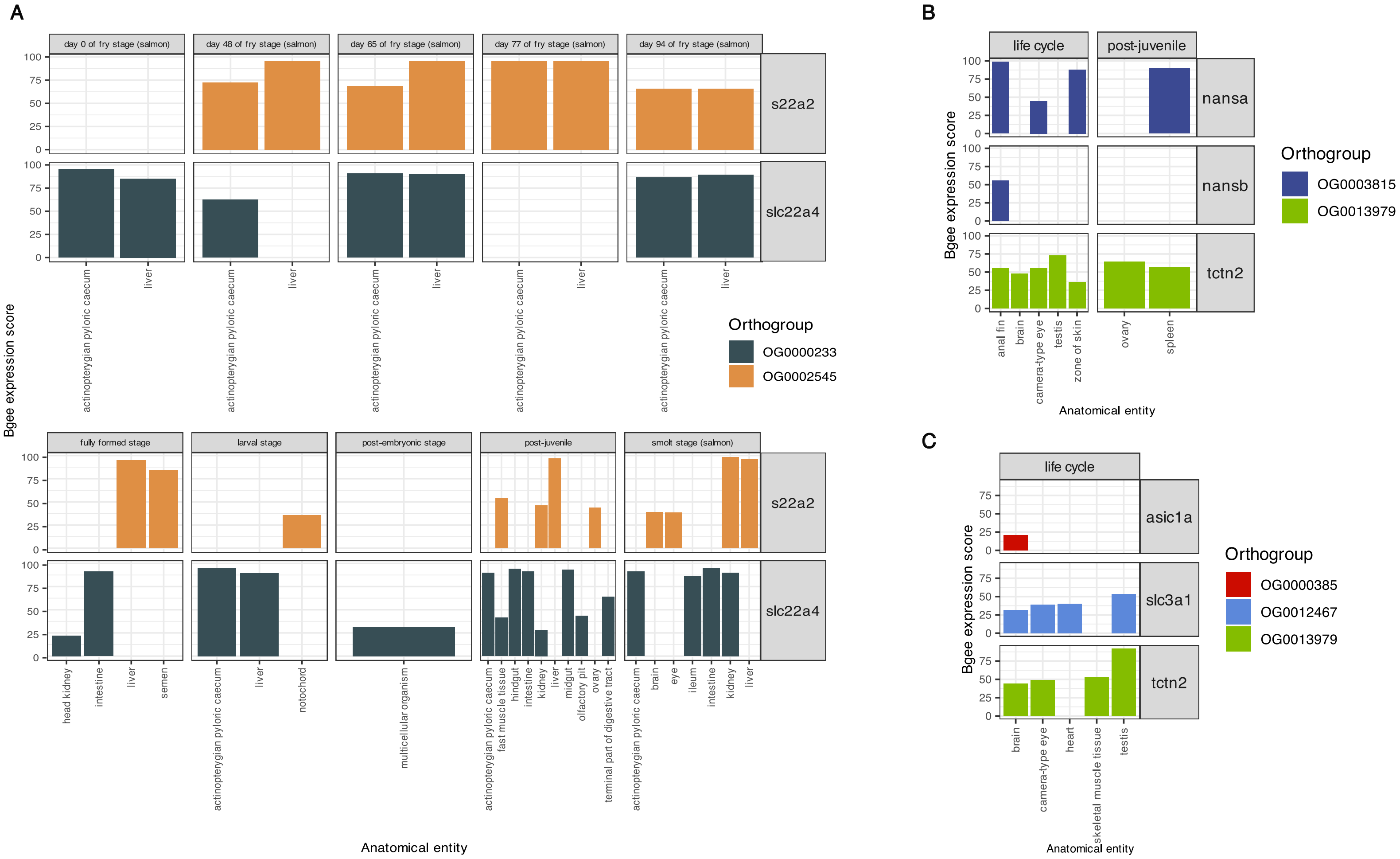

Fig S10: Expression of convergent orthogroups in species harbouring convergent substitutions. Using Bgee expression we show that the convergent genes have a wide spatial and temporal breadth, being expressed across multiple tissues and different developmental stages. (A) Solute-carrier protein *slc22a2*, shows high expression in the liver and pyloric caecum across multiple larval stages of *Salmo salar*, with a subsequent expression in the kidney in the post-juvenile stages. (B) N-acetylneuraminic acid synthase (*NANS*) is highly expressed in the anal fin, skin, and eye of *Astatotilapia calliptera,* as well as in the spleen during the early juvenile stages. We also observed a difference in expression between gene copies, with *nansa* showing high expression across the anal fin, eye, and skin, whereas *nansb* was expressed only in the anal fin. This divergence in expression patterns between paralogs may reflect a partitioning of functional roles across gene copies. (C) Tectonic family member 2, *tctn2* was expressed in multiple tissues in *Neolamprologus brichardi,* with the highest expression in testis. The gene had comparable levels of expression in brain and eye across both Astatotilapia *calliptera* and *Neolamprologus brichardi*, suggesting conserved function.

A

Synchiropus splendidus	Callionymidae	XP_53703524
Doryrhamphus excisus	Syngnathidae	XP_57942490
Dunckerocampus dactyliophorus	Syngnathidae	XP_54611659
Corythoichthys intestinalis	Syngnathidae	XP_57676214
Syngnathus acus	Syngnathidae	XP_37125112
Hippocampus zosterae	Syngnathidae	XP_51902150
Hippocampus zosterae	Syngnathidae	XP_51902860
Periophthalmus magnuspinnatus	Oxudercidae	XP_55082611
Sphaeramia orbicularis	Apogonidae	XP_30009643
Echeneis naucrates	Echeneidae	XP_29375408
Salarias fasciatus	Blenniidae	XP_29956907
Acanthochromis polyacanthus	Pomacentridae	XP_51793947
Amphiprion ocellaris	Pomacentridae	XP_54873016

B

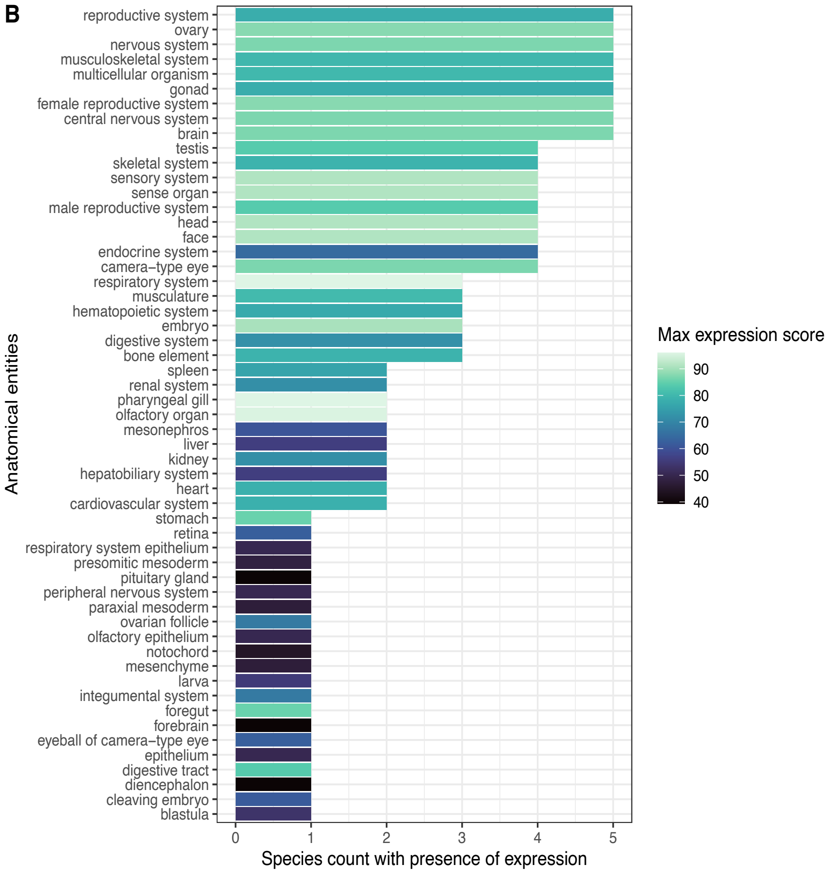

Fig S11: Case of convergent substitution in seven branches. Panel (A) shows the species with harbouring the convergent substitution in the polypeptide N-acetylgalactosaminyltransferase 6 (*GALNTL6*) gene along with their family classification and protein ID respectively. (B) Using data from Bgee we observed high expression in the reproductive and skeletal systems across teleost species. The species with available expression data in this dataset are *Anguilla anguilla*, *Astyanax mexicanus*, *Danio rerio*, *Esox lucius*, and *Salmo salar*.

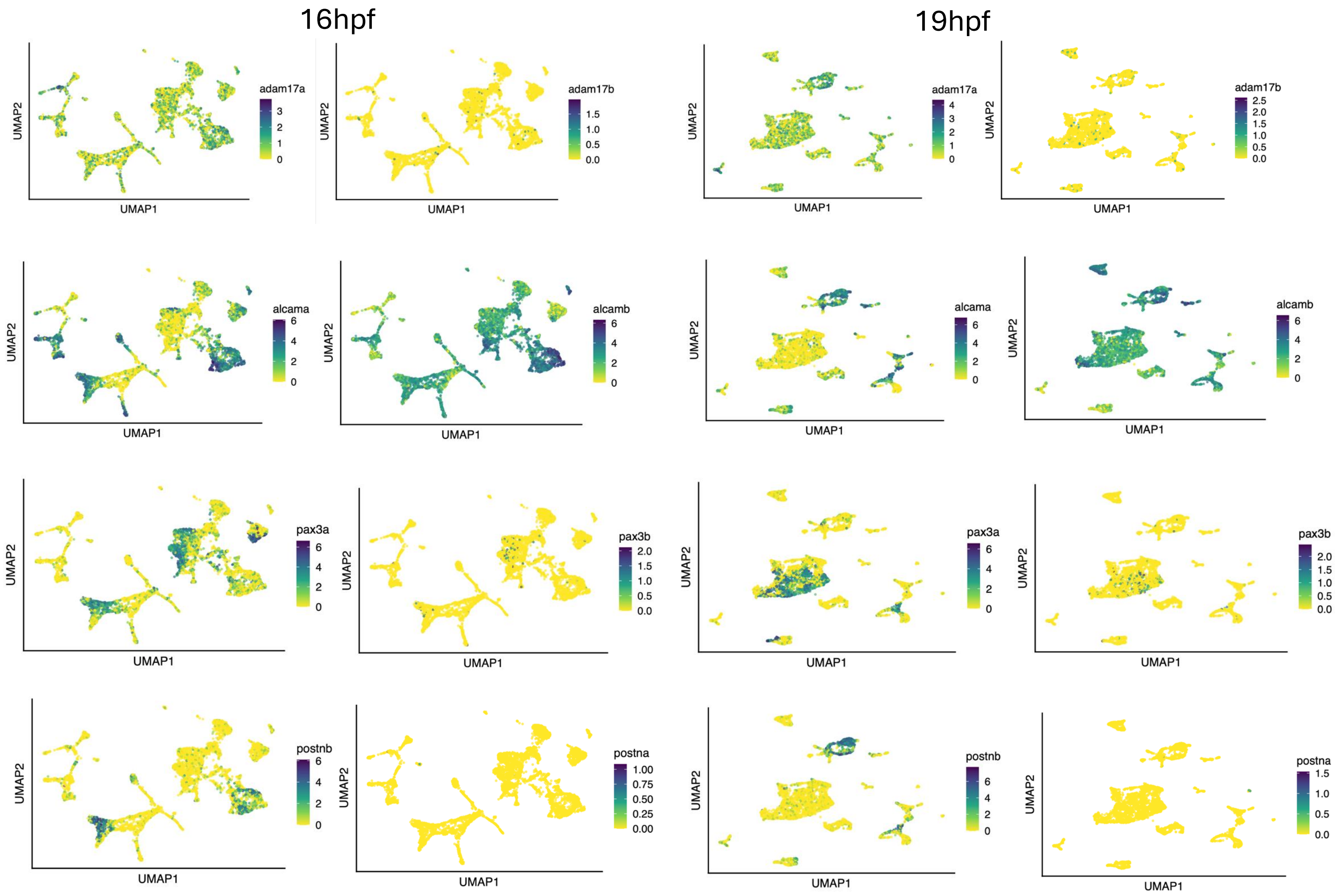

Fig S12: Expression divergence between paralogs. Single-cell RNA-seq data of 16 hours post fertilization and 19 hours post fertilization embryos showing difference in gene expression between paralogs. Similar trend was observed for all other time-points.

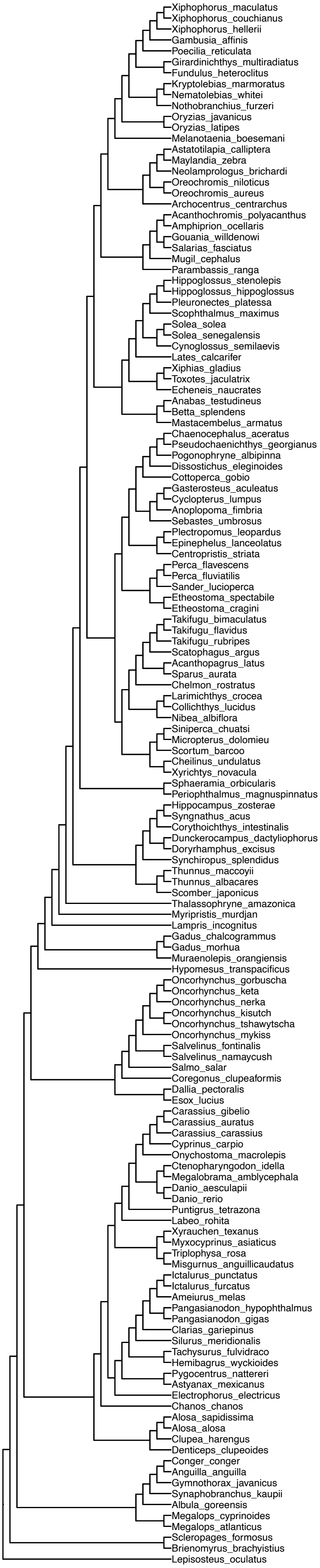
Xiphophorus_maculatus
Xiphophorus_couchianus
Xiphophorus_hellerii
Gambusia_affinis
Poecilia_reticulata
Girardinichthys_multiradiatus
Fundulus_heteroclitus
Kryptolebias_marmoratus
Nematolebias_whitei
Nothobranchius_furzeri
Oryzias_javanicus
Oryzias_latipes
Melanotaenia_boesemani
Astatotilapia_calliptera
Maylandia_zebra
Neolamprologus_brichardi
Oreochromis_niloticus
Oreochromis_aureus
Archocentrus_centrarchus
Acanthochromis_polyacanthus
Amphiprion_ocellaris
Gouania_willdenowi
Salarias_fasciatus
Mugil_cephalus
Parambassis_ranga
Hippoglossus_stenolepis
Hippoglossus_hippoglossus
Pleuronectes_platessa
Scophthalmus_maximus
Solea_solea
Solea_senegalensis
Cynoglossus_semilaevis
Lates_calcarifer
Xiphias_gladius
Toxotes_jaculatrix
Echeneis_naucrates
Anabas_testudineus
Betta_splendens
Mastacembelus_armatus
Chaenocephalus_aceratus
Pseudochaenichthys_georgianus
Pogonophryne_albipinna
Dissostichus_eleginoides
Cottoperca_gobio
Gasterosteus_aculeatus
Cyclopterus_lumpus
Anoplopoma_fimbria
Sebastes_umbrosus
Plectropomus_leopardus
Epinephelus_lanceolatus
Centropristis_striata
Perca_flavescens
Perca_fluviatilis
Sander_lucioperca
Etheostoma_spectabile
Etheostoma_cragini
Takifugu_bimaculatus
Takifugu_flavidus
Takifugu_rubripes
Scatophagus_argus
Acanthopagrus_latus
Sparus_aurata
Chelmon_rostratus
Larimichthys_crocea
Collichthys_lucidus
Nibea_albiflora
Siniperca_chuatsi
Micropterus_dolomieu
Scortum_barcoo
Cheilinus_undulatus
Xyrichtys_novacula
Sphaeramia_orbicularis
Periophthalmus_magnuspinnatus
Hippocampus_zosterae
Syngnathus_acus
Corythoichthys_intestinalis
Dunckerocampus_dactyliophorus
Doryrhamphus_excisus
Synchiropus_splendidus
Thunnus_maccoyii
Thunnus_albacares
Scomber_japonicus
Thalassophryne_amazonica
Myripristis_murdjan
Lampris_incognitus
Gadus_chalcogrammus
Gadus_morhua
Muraenolepis_orangiensis
Hypomesus_transpacificus
Oncorhynchus_gorbuscha
Oncorhynchus_keta
Oncorhynchus_nerka
Oncorhynchus_kisutch
Oncorhynchus_tshawytscha
Oncorhynchus_mykiss
Salvelinus_fontinalis
Salvelinus_namaycush
Salmo_salar
Coregonus_clupeaformis
Dallia_pectoralis
Esox_lucius
Carassius_gibelio
Carassius_auratus
Carassius_carassius
Cyprinus_carpio
Onychostoma_macrolepis
Ctenopharyngodon_idella
Megalobrama_amblycephala
Danio_aesculapii
Danio_rerio
Puntigrus_tetrazona
Labeo_rohita
Xyrauchen_texanus
Myxocyprinus_asiaticus
Triplophysa_rosa
Misgurnus_anguillicaudatus
Ictalurus_punctatus
Ictalurus_furcatus
Ameiurus_melas
Pangasianodon_hypophthalmus
Pangasianodon_gigas
Clarias_gariepinus
Silurus_meridionalis
Tachysurus_fulvidraco
Hemibagrus_wyckioides
Pygocentrus_nattereri
Astyanax_mexicanus
Electrophorus_electricus
Chanos_chanos
Alosa_sapidissima
Alosa_alosa
Clupea_harengus
Denticeps_clupeoides
Conger_conger
Anguilla_anguilla
Gymnothorax_javanicus
Synaphobranchus_kaupii
Albula_goreensis
Megalops_cyprinoides
Megalops_atlanticus
Scleropages_formosus
Brienomyrus_brachyistius
Lepisosteus_oculatus

Fig S13: Phylogenetic tree of species. Phylogenetic tree of 143 teleost fish species used in the study.
